# Supplementary material for: Pain-free resting-state functional brain connectivity predicts individual pain sensitivity
Source: Nat Commun. 2020 Jan 10;11:187. doi: 10.1038/s41467-019-13785-z (PMC6954277; doi:10.1038/s41467-019-13785-z)
Supplement: Supplementary file 1 — Supplementary Information [file 41467_2019_13785_MOESM1_ESM.pdf]

Supplementary Information

Pain-free resting-state functional brain connectivity predicts individual pain sensitivity

Spisak et al.

Supplementary Table 1. Summary statistics of pain sensitivity, in-scanner motion and demographic data.
Summary statistics were computed after exclusions. Abbreviations: BMI: body mass index, FD: framewise displacement, Diff MRI-QST: difference between MRI and QST measurements in days, PSQ: pain sensitivity questionnaire, PCS: pain catastrophising questionnaire, STAI: state-trait anxiety inventory, PSQI: Pittsburgh sleep quality index, ADS-K: German depression scale, PSQ20: perceived stress questionnaire, QST: quantitative sensory testing, pain sens.: pain sensitivity, MAE: mean absolute error.

	Study 1	*Study 2*	*Study 3*
Demographics			
**Site location**	*Bochum, GER*	*Essen, GER*	*Szeged, HUN*
**N before exclusion**	*39*	*48*	*29*
**N after exclusion**	*35*	*37*	*19*
**Age (mean ± sd)**	*26.1 ± 3.9*	*24.9 ± 3.5*	*24.8 ± 3.1*
**Age (min, max)**	*20, 39*	*19, 36*	*21, 31*
**Sex (%female)**	*37%*	*54%*	*53%*
**BMI (mean ± sd)**	*23.4 ± 2.3*	*23.2 ± 2.7*	*23.1 ± 4.3*
Confounders - MRI			
**meanFD (mean ± sd)**	*0.08 ± 0.02mm*	*0.07 ± 0.02mm*	*0.07 ± 0.02mm*
**meanFD (min, max)**	*0.04, 0.13*	*0.04, 0.12*	*0.04, 0.12*
**%scrubbed (mean ± sd)**	*7.1 ± 7.4%*	*6.4 ± 6.7%*	*8.4 ± 8.0%*
**%scrubbed (min, max)**	*0, 26%*	*0, 29%*	*2, 26%*
**Diff MRI-QST (mean ± sd)**	*0 ± 0 days*	*3.1 ± 2.9 days*	*2.3 ± 1.4 days*
**Diff MRI-QST (min, max)**	*0, 0 days*	*1, 18 days*	*1, 5 days*
Confounders - questionnaires			
**PSQ pain sensitivity (mean ± sd)**	*56.6 ± 19.1*	*55.3 ± 18.5*	*44.7 ± 14.8*
**PCS catastrophising (mean ± sd)**	*15.47 ± 7.9*	*14.9 ± 9.6*	*9.8 ± 10.4*
**STAI state anxiety (mean ± sd)**	*42.8 ± 4.0*	*33.4 ± 7.4*	*31.6 ± 10.2*
**STAI trait anxiety (mean ± sd)**	*44.4 ± 3.1*	*37.2 ± 9.9*	*31.2 ± 8.6*
**PSQI sleep quality (mean ± sd)**	*N/A*	*6.1 ± 3.1*	*3.0 ± 2.2*
**ADS-K depression (mean ± sd)**	*7.0 ± 4.1*	*7.8 ± 6.6*	*5.8 ± 5.9*
**PSQ20 stress (mean ± sd)**	*N/A*	*44.4 ± 9.4*	*26.5 ± 4.0*
Prediction target			
**QST pain sens. (mean ± sd)**	*0.008 ± 0.73*	*0.29 ± 0.80*	*-0.44 ± 0.46*
**QST pain sens. (min, max)**	*-1.45, 1.53*	*-1.82, 1.57*	*-1.2, 0.56*
Prediction			
**Explained variance**	*39.2%*	*18.1%*	*17.0%*
**MAE**	*0.271*	*0.553*	*0.248*
**perm. P-value MAE**	*>0.0001*	*0.039*	*0.025*
**MSE**	*0.316*	*0.536*	*0.173*
**perm. P-value MSE**	*>0.0001*	*0.02*	*0.025*
**Pearson's r**	*0.63*	*0.43*	*0.47*
**Perm. P-value**	*>0.0001*	*0.0037*	*0.021*
**Parametric p-value**	*0.00006*	*0.008*	*0.04*

Supplementary Table 2 Recruitment numbers and exclusions per criteria

For privacy reasons, possible cases with incidental findings are not shown in the table.

	Study 1	Study 2	Study 3	*Total*
Recruited	39	48	29	116
Excluded due to extreme QST values	-0	-3	-2	-5
Excluded due to motion	-4	-8	-8	-20
Eligible for analysis	35	37	19	91

Supplementary Table 3. Results of confounder analysis not shown in main Table 1.

Trait anxiety and blood pressure (systolic and diastolic) as measured on the day of QST measurement.

	anxiety trait		BP sys. QST		BP dias. QST	
	R^2	p	R^2	p	R^2	p
Study 1	0.000	1.000	N/A	N/A	N/A	N/A
Study 2	0.001	0.876	0.010	0.547	0.002	0.816
Study 3	0.040	0.412	0.013	0.723	0.110	0.291

Supplementary Table 4. Predictive connections of the RPN signature with bootstrapped confidence intervals and p-values.

Non-zero regression coefficients naturally delineate the predictive sub-network. Regions and corresponding large-scale resting-state network (RSN) modules are to be interpreted as in the MIST atlas (see Methods, original atlas-index is given). Predictive connections are ordered by their absolute predictive weights. Connectivity strengths associated with higher and lower sensitivity to pain are highlighted in red and blue, respectively. The 95% confidence intervals (ci) and the p-values are based on bootstrapping analysis. Note that the bootstrap analysis only allows for inference conditional on variable selection. Abbreviations: CER: cerebellum, roman numbers: cerebellar lobes, GM grey matter, VAN: ventral attention network, SN: salience network, BG: basal ganglia, Thal: thalamus, Hb: habenula, MLN: mesolimbic network, FPN: frontoparietal network, SMN: somatomotor network, DMN: default mode network, VN: visual network, Ins: insula, PO: parietal operculum, SII: secondary somatosensory cortex, STG: superior temporal gyrus, FEF: frontal eye-field, PrCG: precentral gyrus, PoCG: postcentral gyrus, SMC: supplementary motor cortex, Put: putamen, Caud: caudate nucleus, Acc: nucleus accumbens, LOG: lateral orbital gyrus, CF: collateral fissure, OTG: occipitotemporal gyrus, MFG: middle frontal gyrus, IPS: intraparietal sulcus, pgACC: perigenual anterior cingulate cortex, PrC: precuneus cortex. Prefix: L: left, R: right, a: anterior, p: posterior, v: ventral, d: dorsal, l: lateral, m: medial.

Predictive connections between brain regions						weight	95% ci	p
**region**	**RSN**	**idx**	**region**	**RSN**	**idx**			
PO/pSTG	VAN+SN+BG+Thal	119	pPut	VAN+SN+BG+Thal	25	0.270	[0.06, 0.38]	<0.0001
FP	FPN	75	5	CER	48	0.245	[0.06, 0.37]	<0.0001
pCVI	CER	9	SMC	VAN+SN+BG+Thal	28	-0.200	[-0.37, -0.02]	0.001
R aCrus2	CER	62	lPrCG	SMN	93	0.150	[0.02, 0.3]	0.0001
dPrCG	SMN	67	pmVN	VN	51	-0.102	[-0.3, -0.006]	0.003
pdlVN	VN	43	mVN	VN	40	0.095	[0.004, 0.21]	0.01
L IPL	DMN	114	mean GM	mean GM	-	-0.086	[-0.2, -0.004]	0.009
vCaud	VAN+SN+BG+Thal	2	plVN	VN	39	0.085	[0.001, 0.236]	0.02
Acc	MLN	78	pvmVN	VN	107	-0.073	[-0.24, -0.004]	0.006
CF	MLN	79	vlPrCG	SMN	110	-0.062	[-0.24, -0.004]	0.007
5	CER	48	pdlVN	VN	43	-0.059	[-0.2, -0.002]	0.02
pThal/Hb	VAN+SN+BG+Thal	36	plVN	VN	39	0.058	[-0.009, 0.22]	0.03
dCVI	CER	44	lOTG	FPN	117	-0.057	[-0.19, -0.002]	0.01
dCiX	CER	11	L vMFG	FPN	105	-0.056	[-0.22, -0.001]	0.01
R IPS	FPN	20	plVN	VN	39	-0.054	[-0.17, 0.005]	0.03
avIns	VAN+SN+BG+Thal	12	admVN	VN	19	-0.044	[-0.21, 0]	0.02
R aMFG	FPN	58	lPoCG	VAN+SN+BG+Thal	102	0.043	[-0.1, 0.13]	0.3
CrusI	CER	84	dPoCG	VAN+SN+BG+Thal	116	-0.017	[-0.15, 0.03]	0.1
pgACC	DMN	115	mSTG	VAN+SN+BG+Thal	88	0.009	[-0.04, 0.11]	0.2
Precun	DMN	103	LOG	MLN	109	-0.003	[-0.16, 0.06]	0.1
vThal	VAN+SN+BG+Thal	36	FEF	VAN+SN+BG+Thal	113	-0.001	[-0.11, 0.1]	0.3

Supplementary Figure 1. Nipype workflow of the MRI image preprocessing and connectivity calculation for the RPN-signature.
Workflow is based on the in-house PUMI workflow library system (*https://github.com/spisakt/PUMI*).
Source code of the workflow:
https://github.com/spisakt/PAINTeR/blob/master/pipeline/pipeline_PAINTeR.py
High resolution workflow graph is available on-line:
https://github.com/spisakt/PAINTeR/blob/master/pipeline/graph.png

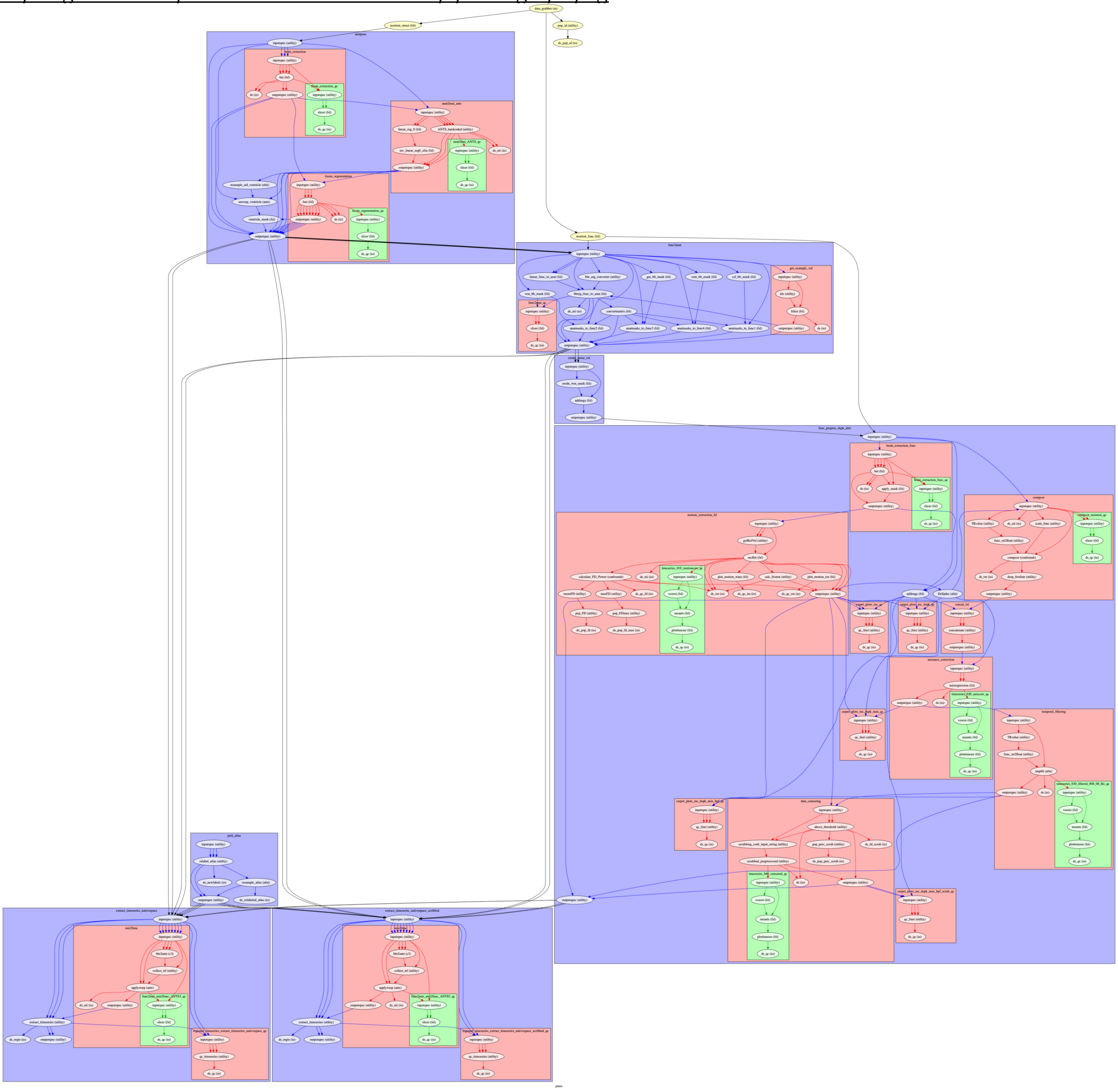

Supplementary Figure 2. Quality-check images for the spatial standardisation of the anatomical images in Study 1.

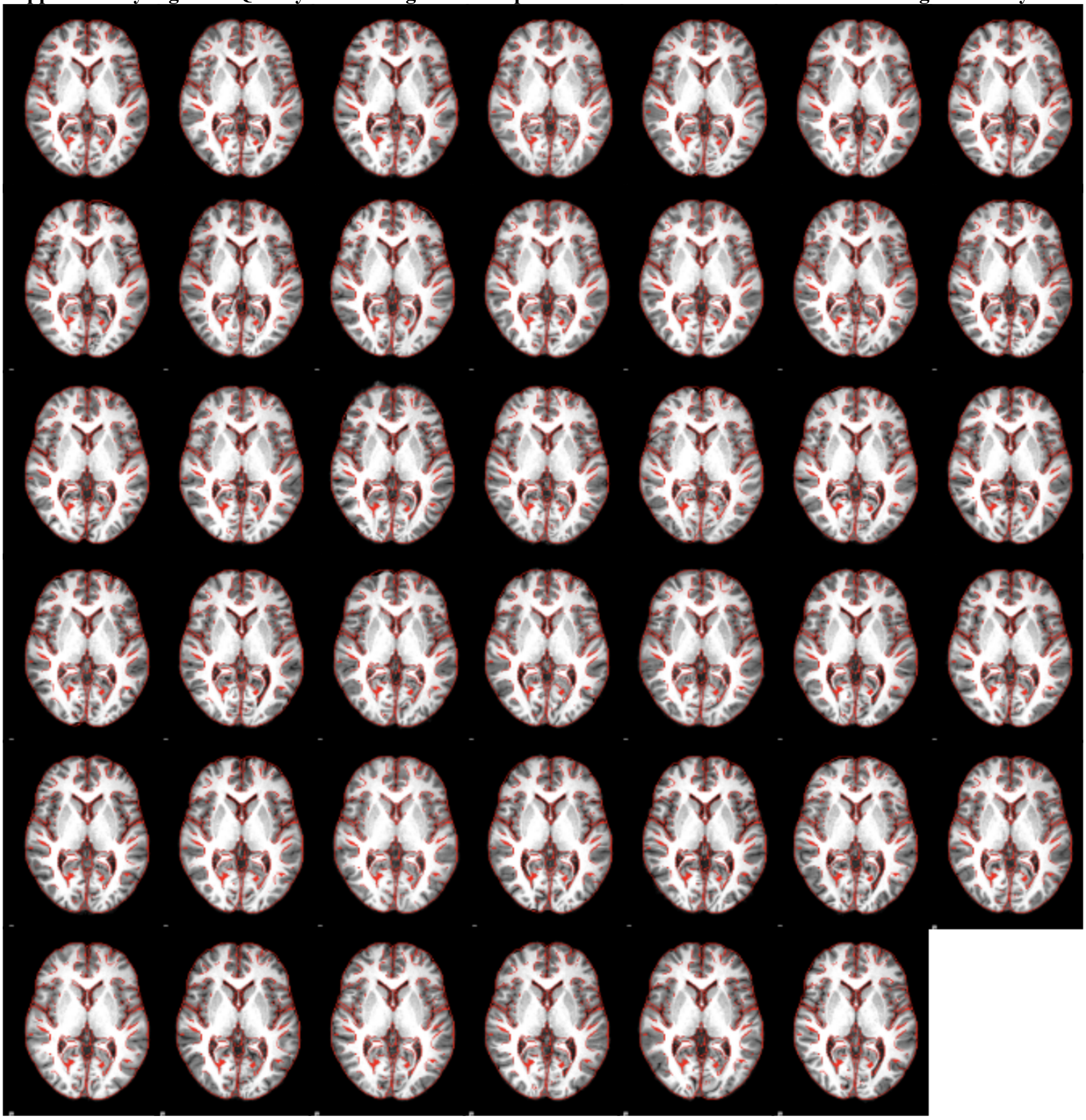

Supplementary Figure 3. Quality-check images for the spatial standardisation of the anatomical images in Study 2.

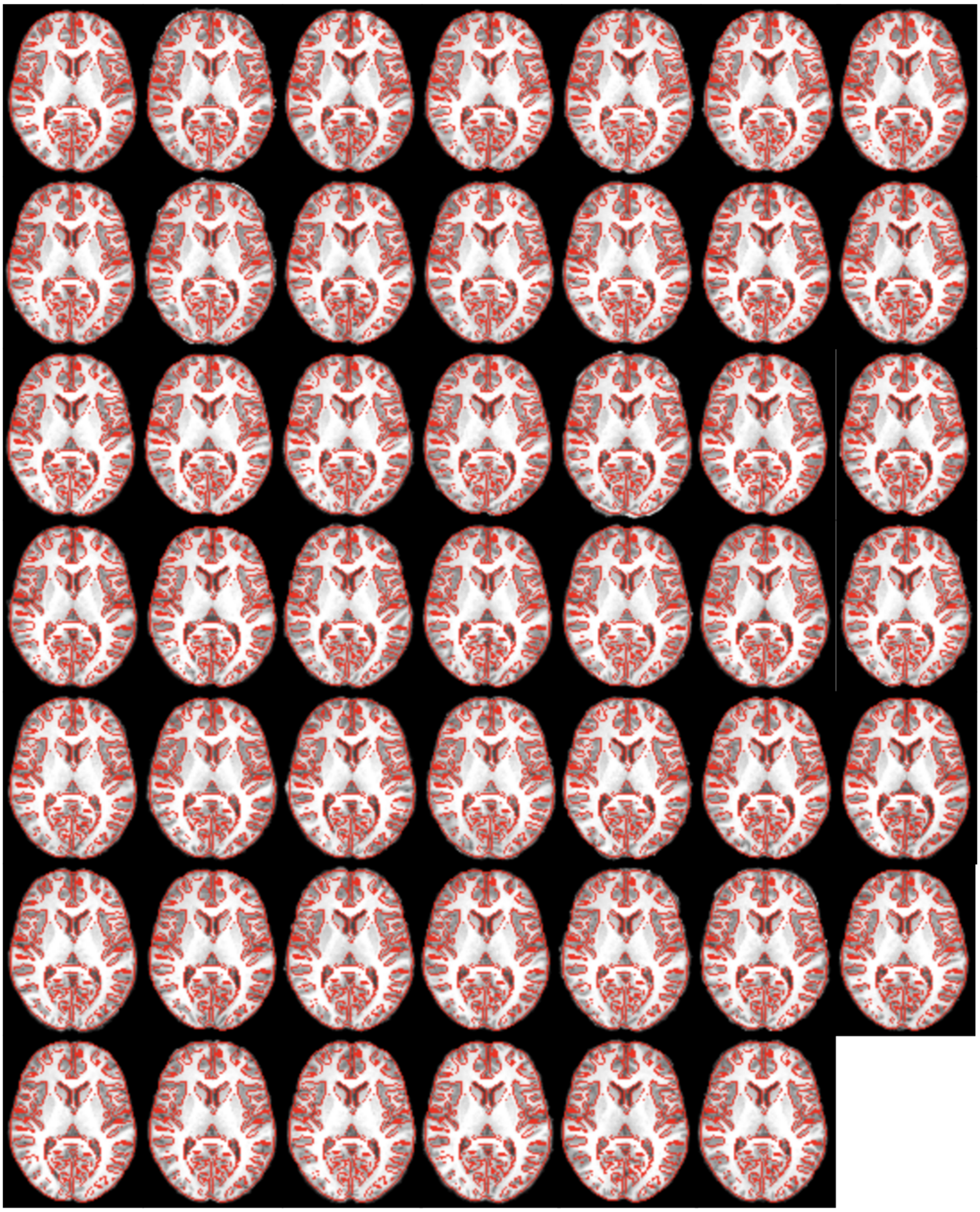

Supplementary Figure 4. Quality-check images for the spatial standardisation of the anatomical images in Study 3.

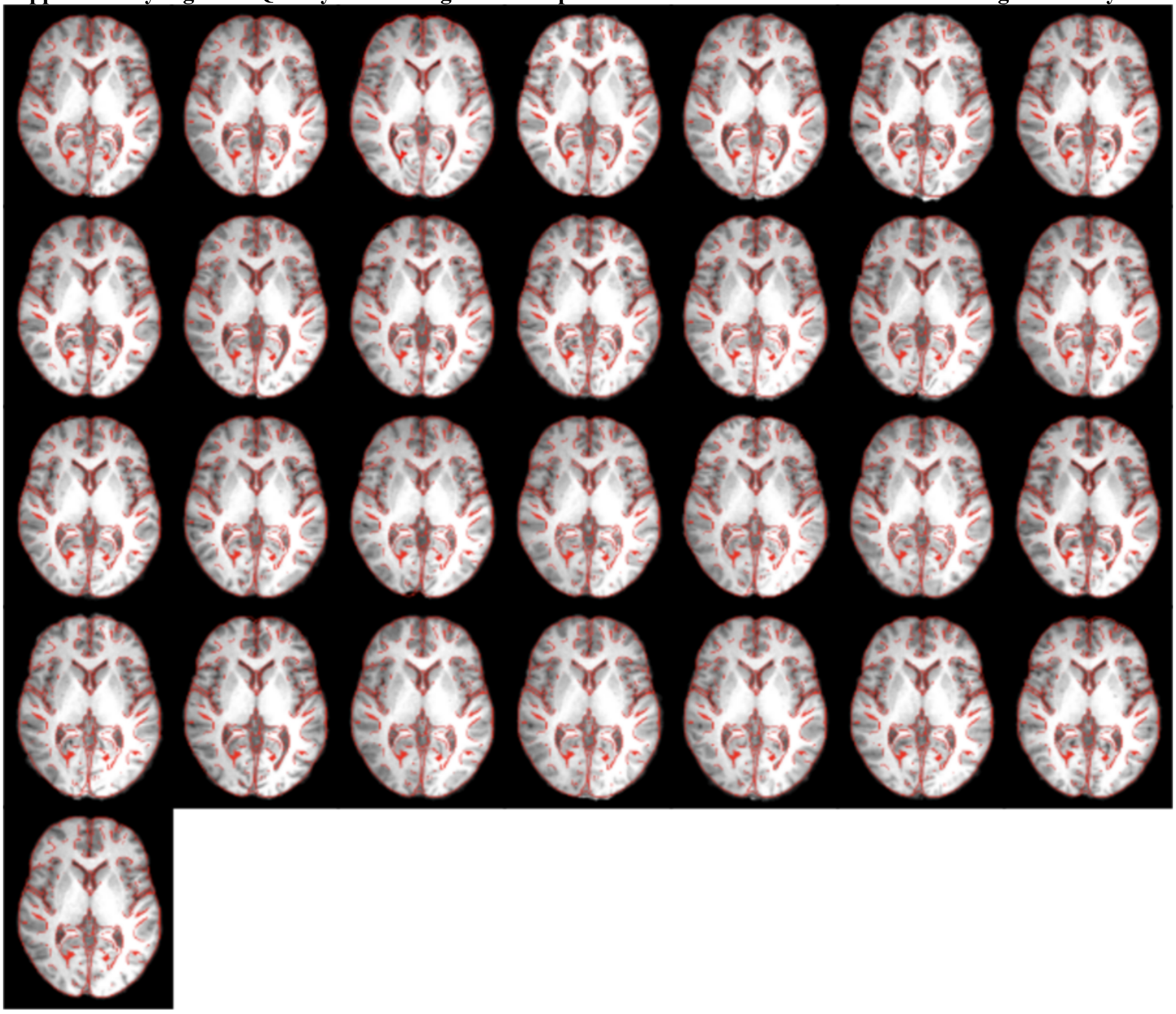

Supplementary Figure 5. Illustration of the brain atlas individualization procedure and the construction of study-specific probability maps for the Putamen region with 6 example subjects.

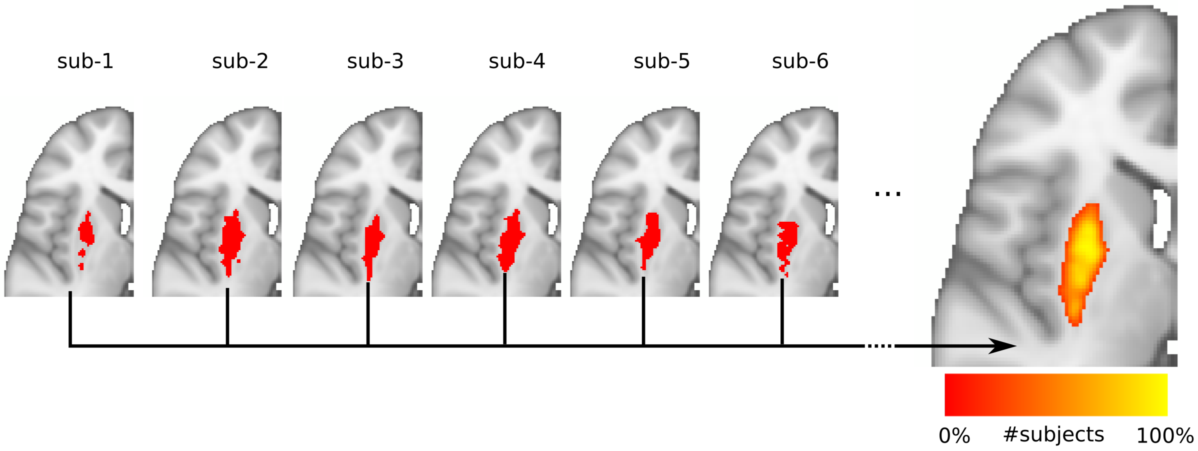

Supplementary Note 1

November 19, 2019

1 Analysis of the rationale beyond using the composite score of (Zunhammer et al., 2016) as a prediction target for the RPN-signature.

1.1 *Supplementary Analysis 1 for the paper "Pain-free resting-state functional brain connectivity predicts individual pain sensitivity"*

Supplementary Analyses 1 is also available at the following link:

https://raw.githack.com/spisakt/RPN-signature/master/notebooks/Supplementary_Analysis_1.html

Alternatively, to edit and run the code:

https://nbviewer.jupyter.org/github/pni-lab/RPN-signature/blob/master/notebooks/Supplementary_Analysis_[illegible]
>By clicking on "Execute on Binder" in the top right corner, the reviewers can enter the interactive mode where the code of the analysis can be edited and run in a dedicated python environment.

2 Introduction

In statistics, and particularly, in biomedical research, composite scores are commonly calculated from multiple variables in order to form simple, reliable and valid measures of latent, theoretical constructs (Song et al., 2013, Babbie, 2016).

A common approach is, for instance, to convert each variable to a z-score and then unit-weight the variables (i.e., take a simple sum of z-scores). This represents an equal weighting that controls for the fact that the variables are on different metrics (Bobko et al, 2007).

Such and similar composite scores are frequently used in pain research, as well, e.g. to describe the quality of pain (Victor et al., 2008), intensity of postsurgical (Jensen et al., 2002) and chronic pain (Jensen et al., 1999), disease activity (Haugen et al., 2010), or pain sensitivity (O'Neill, 2014, Starkweather et al., 2016, Zunhammer et al., 2016). The benefit of such scores is in their ability to capture multiple aspects of interest into a single numerical value, which might increase the face and construct validity and sensitivity to change (Haugen et al., 2010). Although these scores are mostly based on well-established and validated measurement protocols like the Quantitative Sensory Testing (QST, Rolke et al., 2006) (Jensen et al., 2002, Jensen et al., 1999, O'Neill, 2014,Starkweather et al., 2016, Zunhammer et al., 2016), the composition of these scores depends on the specific aims of studies.

For instance, the QST-based pain sensitivity score proposed in (Zunhammer et al., 2016) is a unit weighted measure of the z-score transformed pain thresholds assessed in three different sensory modalities, namely heat, cold and mechanical pain thresholds (HPT, CPT and MPT, respectively). This composite score was shown to be associated to combined glutamate and glutamine levels in pain processing brain regions and importantly, it was found to *outperform* the single modalities it is based on (Zunhammer et al., 2016, Supplemental Digital Content 3).

While this indirect evidence suggests that this pain sensitivity score might be a useful measure of the modality-independent component shaping one's sensitivity to pain, a thorough characterization of the the effectiveness of this composite score still needs to be done, before using it as gold-standard proxy variable for modality-independent pain sensitivity e.g. in predictive modeling studies.

3 Aim

In this python notebook, we perform a *multi-stage* analysis to investigate the rationale of using the composite pain sensitivity score as defined by (Zunhammer et al., 2016) for predictive modeling purposes like the *RPN-signature*.

4 Overview of the analysis

Most of the composite scales are assumed by their developers and users to be primarily a measure of *one latent variable*. When it is also assumed that the scale conforms to the effect indicator model of measurement (as is almost always the case in psychological assessment), it is important to support such an interpretation with evidence regarding the internal structure of that scale (Zinbarg et al., 2006).

After importing necessary python modules and loading the data, here we perform four stages of analysis, addressing four key question: - **Question Q1.** Is there a common, modality-independent component shared across the investigated pain modalities? - **Question Q2.** Do the the composite score of (Zunhammer et al., 2016) capture this putative, shared, modality-independent component? - **Question Q3.** Do we have evidence that the prediction of the RPN-signature (the RPN-score, trained using the the score of (Zunhammer et al., 2016)) captures the shared, modality-independent component? - **Question Q4.** To what extent is the RPN-score biased towards any of the modalities? Is its correlation with the single thresholds lower than expected from its correlation to the composite score of (Zunhammer et al., 2016)?

5 Import neccessary modules and load data.

[13]:
```
import pandas as pd
import numpy as np
import numpy as np
import matplotlib.pyplot as plt
import networkx as nx
import seaborn as sns; sns.set()
```

```
sns.set(style="ticks")
import warnings
warnings.filterwarnings('ignore')
from mlxtend.evaluate import permutation_test
from sklearn.decomposition import PCA
from sklearn.preprocessing import RobustScaler
from tqdm import tqdm
import statsmodels.api as sm

# Load all data:
study1 = pd.read_csv("../res/bochum_sample_excl.csv")[["HPT","CPT",
                                                       "MPT_log_geom",
                                                      ␣
 ↪"mean_QST_pain_sensitivity",
                                                       "prediction"]]
study2 = pd.read_csv("../res/essen_sample_excl.csv")[["HPT", "CPT",
                                                      "MPT_log_geom",
                                                     ␣
 ↪"mean_QST_pain_sensitivity",
                                                      "prediction"]]
study3 = pd.read_csv("../res/szeged_sample_excl.csv")[["HPT", "CPT",
                                                       "MPT_log_geom",
                                                      ␣
 ↪"mean_QST_pain_sensitivity",
                                                       "prediction"]]
# merge datasets
study1["study"]="Study 1"
study2["study"]="Study 2"
study3["study"]="Study 3"
df = pd.concat([study1, study2, study3])
df = df.dropna()
```

6 Question Q1: Is there a common, modality-independent component shared across the investigated pain modalities?

We invetsigate heat, cold and mechanical pain thresholds (HPT, CPT and MPT, respecuvely), as measured via Quantitative Sensory Testing (QST) with the aim of identifying any "modality-independent" component, shared across the these measurements.

Specifically, we assume that all three variables consist of three compomnents: - A modality-specific component (different for heat, cold and mechanical stimuli). The assumption of such a modality-specific component is supported by a wealth of previous research suggesting that different biological systems underly the three threshold types. Heat, cold and mechanical pain stimuli are known to be picked up by different sets of nocisensors within nociceptive neurons (Dubin & Patapoutian, 2010). Nerve-block studies further have suggested that the three different types of pain thresholds rely on different sets of nociceptive neurons: HPT mainly depends on C-fibers, whereas MPT mainly

depend on A-delta-fibers, while the relative contribution of C- and Ad-fibers to cold pain thresholds is less clear (Rolke et al., 2006). - A modality-independent component (shared between heat, cold and mechanical stimuli). Here we test the presence and significance of this component. - A “noise” component (e.g. confounds specific for the different measures, like reaction time confounds, skin physiology, etc.)

Of note, the experimental procedures for HPT and CPT are may likely be more similar compared to the MPT in this respect. Procedures for the thermal thresholds differ only in the direction of temperature changes and in the wording of instructions; both HPT and CPT are obtained with an automated thermode and based on a method of ascending limits. In contrast, the MPT is obtained with hand-held pin-pricks and based on a staircase method and involves more stimulus repetitions. Moreover, thermal and mechanical thresholds may be affected by different types of skin physiology may add variance to these measures. This “noise” component has the potential to partly mask the common- and modality-specific components.

6.1 Step 1. McDonald’s omega

McDonald’s Omega as a measure of internal consistency. It measures whether several items that propose to measure the same general construct produce similar scores.

Possible outcomes and interpretations: The value of McDonald’s Omega falls between 0 and 1. While there are rules of thumb for cut-off values, those can be misleading and are more appropriate for variables which are meant to measure exactly the same latent variable (Dunn et al., 2014). In general, larger values mean stronger internal consistency and a value below 0.5 indicates the lack of internal consistency across the variables.

R-code:

```
# load data into data.frame called df
library(psych)
omega(df)
```

6.1.1 Results

Omega (‘omega total’) was found to be **0.62**.

6.1.2 Interpretation

This value suggest internal consistency across the investigated pain threshold measures.

To gain a more detailed insight into the consistency structure, below, we investigate: - the correlations between the variables (Step 2), - and the underlying latent components by means of principal component analysis (Step 3).

6.2 Step 2. Correlation analysis

Below we compute the correlation between all possible pairs of pain thresholds, as well as the mean correlation across all possible pairs. P-values are calculated via permutation testing.

[16]:

```
#pairplot with histograms:
#sns.pairplot(df[["HPT", "CPT", "MPT_log_geom", "study"]], kind="reg")
# tip: add hue="study", to see data separately for each study

# Plot correlations
fig, (ax1, ax2, ax3) = plt.subplots(ncols=3, figsize=(15,5))
sns.regplot(x=df['HPT'], y=df['CPT'], ax=ax1)
ax1.set_xlabel('HPT (°C)')
ax1.set_ylabel('CPT (°C)')
sns.regplot(x=df['HPT'], y=df['MPT_log_geom'], ax=ax2)
ax2.set_xlabel('HPT (°C)')
ax2.set_ylabel('MPT (log mN)')
sns.regplot(x=df['CPT'], y=df['MPT_log_geom'], ax=ax3)
ax3.set_xlabel('CPT (°C)')
ax3.set_ylabel('MPT (log mN)')
ran=np.max(df['CPT'])-np.min(df['CPT'])
plt.xlim(np.min(df['CPT'])-0.1*ran, np.max(df['CPT'])+0.1*ran)
ran=np.max(df['MPT_log_geom'])-np.min(df['MPT_log_geom'])
ax3.set_ylim(np.min(df['MPT_log_geom'])
                         -0.1*ran,np.max(df['MPT_log_geom'])+0.1*ran)

p_value = permutation_test(df['HPT'], df['CPT'],
                           method='approximate',
                           func=lambda x, y: -np.corrcoef(x, y)[1][0],
                           num_rounds=10000,
                           seed=0)
print("cor(HPT, CPT) = " + str(np.corrcoef(df['HPT'], df['CPT'])[0,1]))
print("Permuation-based p = " + str(p_value))

p_value = permutation_test(df['HPT'], df['MPT_log_geom'],
                           method='approximate',
                           func=lambda x, y: np.corrcoef(x, y)[1][0],
                           num_rounds=10000,
                           seed=0)
print("cor(HPT, MPT_log_geom) = " +
       str(np.corrcoef(df['HPT'], df['MPT_log_geom'])[0,1]))
print("Permuation-based p = " + str(p_value))

p_value = permutation_test(df['CPT'], df['MPT_log_geom'],
                           method='approximate',
                           func=lambda x, y: -np.corrcoef(x, y)[1][0],
                           num_rounds=10000,
                           seed=0)
print("cor(CPT, MPT_log_geom) = " +
       str(np.corrcoef(df['CPT'], df['MPT_log_geom'])[0,1]))
print("Permuation-based p = " + str(p_value))
```

```
p_value = permutation_test(df['HPT'], df['CPT'],
                method='approximate',
                func=lambda x, y: np.mean(np.abs([np.corrcoef(x, y)[0,1],
                                np.corrcoef(x, df['MPT_log_geom'])[0,1],
                                np.corrcoef(y, df['MPT_log_geom'])[0,1]])),
                num_rounds=1000, #set to 10000 for more accuracy
                seed=0)
print("Mean correlation: " +
       str(np.mean(np.abs([np.corrcoef(df['HPT'], df['CPT'])[0,1],
                    np.corrcoef(df['HPT'], df['MPT_log_geom'])[0,1],
                    np.corrcoef(df['CPT'], df['MPT_log_geom'])[0,1]])
    )))
print("Permuation-based p = " + str(p_value))
```

```
cor(HPT, CPT) = -0.5063125884777612
Permuation-based p = 0.0
cor(HPT, MPT_log_geom) = 0.19267290368321235
Permuation-based p = 0.0336
cor(CPT, MPT_log_geom) = -0.06731140025472557
Permuation-based p = 0.2651
Mean correlation: 0.2554322974718997
Permuation-based p = 0.0
```

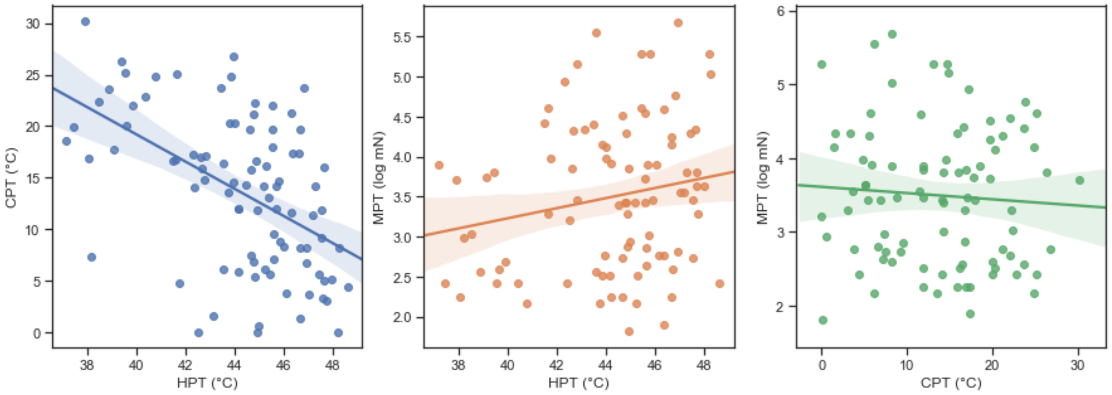

6.2.1 *Supplementary Figure 6. Correlations of CPT, HPT and MPT with each other in all studies.*

Units are °C for CPT and HPT and log(mN) for MPT. The correlation between HPT and CPT and HPT and MPT was significant (RHPT,CPT=-0.51, pHPT,CPT<0.0001, RHPT,MPT=0.19, pHPT,MPT=0.03). The mean correlation across all three modalities was also significant (Rmean=-0.25, pHPT,CPT<0.0001).

6.2.2 Results

Two out of the three possible correlations (HPT-CPT and HPT-MPT) are statistically significant.

The mean correlation is statistically significant, as well, p<0.001.

Below, we plot the correlation structure as a network to aid interpretation.

[18]:
```
# Calculate the correlation between individuals.
# We have to transpose first, because the corr function calculate
# the pairwise correlations between columns.
corr = df[["HPT", "CPT", "MPT_log_geom", "study"]].corr()
corr = corr.where(np.triu(np.ones(corr.shape)).astype(np.bool))

# Transform it in a links data frame (3 columns only):
links = corr.stack().reset_index()
links.columns = ['var1', 'var2','value']
links['value']=np.round(links['value'], decimals=2)

# Remove self correlation (cor(A,A)=1)
links_filtered=links.loc[ (links['var1'] != links['var2']) ]

# Build your graph
G=nx.from_pandas_dataframe(links_filtered, 'var1', 'var2', edge_attr='value')

significant = [(u, v) for (u, v, d) in G.edges(data=True)
                                        if np.abs(d['value']) > 0.15]
significant_value = [d['value'] for (u, v, d) in G.edges(data=True)
                                        if np.abs(d['value']) > 0.15]
nonsignificant = [(u, v) for (u, v, d) in G.edges(data=True)
                                        if np.abs(d['value']) <= 0.15]
nonsignificant_value = [d['value'] for (u, v, d) in G.edges(data=True)
                                        if np.abs(d['value']) <= 0.15]

# Plot the network:
pos = nx.circular_layout(G)
plt.figure(3,figsize=(7,5))
nx.draw(G, pos, with_labels=True, node_color='lightgray', node_size=4000,
         edge_color='lightgray', linewidths=10, width=10, style='dotted')
edges=nx.draw_networkx_edges(G, pos, edge_color=significant_value,
                             edgelist=significant, width=10,
                             edge_cmap=plt.cm.bwr, edge_vmin=-1, edge_vmax=1)

plot=nx.draw_networkx_edge_labels(G,
                             pos,
                             edge_labels=nx.get_edge_attributes(G,'value'),
                             font_color='black',
                             font_size=25)
```

```
colorbar=plt.colorbar(edges)
```

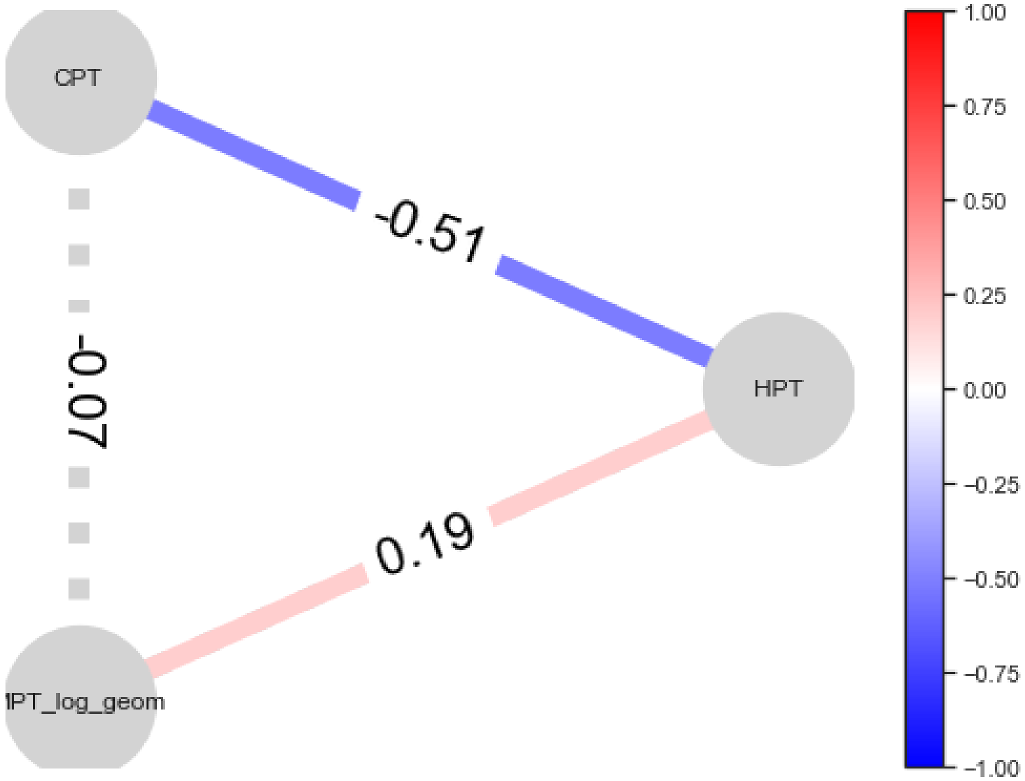

6.2.3 *Supplementary Figure 7. Network representation of the correlation structure across CPT, HPT and MPT.*

Dotted lines and light gray color mean no significance. Solid lines mean significant correlation and are color-coded, according to the legend.

As shown by this plot, the correlation structure of the pain threshold data suggests that: - there is a relationship (or shared component) between the thermal thresholds (HPT and CPT), implied by a strong statistically significant correlation. - there is also a relationship (or shared component) between HPT and MPT, implied by a weaker, but still statistically significant correlation.

6.2.4 Interpretation

Two interpretations are possible: - There **is** a single *shared component across all three modalities*, but we were unable to detect the CPT-MPT relationship in the current sample (e.g. because it is "abolished" by the modality-specific and noise components, which are of different magnitude

in various modalities) - There is **no** shared component between CPT and MPT, but there is one shared component between HPT and CPT and another between HPT and MPT.

The current analysis is unable to decide between the above options.

Solution: unsupervised dimension reduction analysis (Step 3).

6.3 Step 3. Principal Component Analysis

We perform a Principal Component Analysis (PCA) and investigate the contribution ("loadings") of each modality to the principal components of the data in order to determine, which interpretation from above fits the data better. Significance of contributions will be investigated by a permutations test.

Possible outcomes and interpretations:

- If we find a principal component that holds significant contributions from all three variables, it can be considered as an evidence for a common shared component.
- If, to all of the principal components, there is at least one modality that does not have a significant contribution, then there is, most probably, no component that is shared across *all three* modalities, or the variance explained by this component is too small to be captured by PCA.

Below we perform a PCA on the original (unpermuted) data and plot the contribution-matrix (loadings).

[19]:
```
scaler = RobustScaler()
df_pca=df[["HPT", "CPT", "MPT_log_geom"]]
df_pca.loc[:,'HPT'] *= -1  # to align directions of scsales
df_pca.loc[:,'MPT_log_geom'] *= -1  # to align directions of scsales
data_rescaled = scaler.fit_transform(df_pca)
pca = PCA(n_components=3)
pca.fit(data_rescaled)
principal_components=pca.transform(data_rescaled)
loadings = pca.components_.T * np.sqrt(pca.explained_variance_)

plt.matshow(np.abs(loadings),cmap='viridis')
plt.xticks([0,1,2],['PC1','PC2','PC3'],fontsize=10, rotation=65,ha='left')
plt.colorbar()
plt.yticks(range(len(df[["HPT", "CPT", "MPT_log_geom"]].columns.values)),
           df[["HPT", "CPT", "MPT_log_geom"]].columns.values)
#plt.tight_layout()
original_loadings=loadings
pd.DataFrame(np.abs(original_loadings),
    columns=['PC1','PC2','PC3'],
    index=["HPT", "CPT", "MPT"])
```

[19]:

	PC1	PC2	PC3
HPT	0.745046	0.086474	0.239769

```
CPT  0.424109  0.158420  0.385858
MPT  0.221024  0.595476  0.067835
```

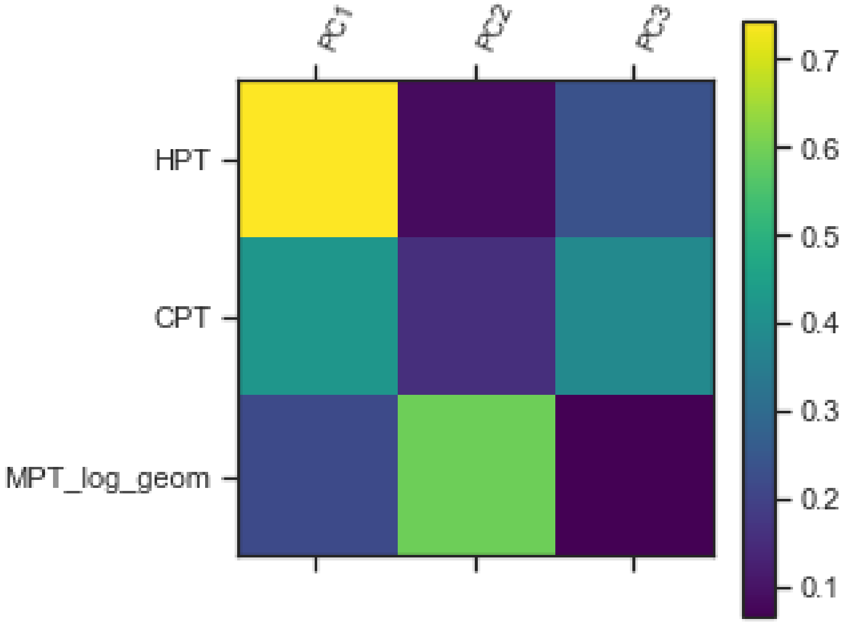

6.3.1 *Supplementary Figure 8. Contribution (loadings) of HPT, CPT and MPT to the principal components.*

Contributions are color-coded according to the color-bar on the left. Loading values are given in the table.

Principal component 1 (PC1) is obviously driven by the thermal thresholds (HPT and CPT), however it is of question, whether the contribution of MPT to PC1 is significant.

Therefore, we permute MPT and repeat the PCA many times to construct a p-value for the null hypothesis of PC1 being independent of MPT.

[22]:
```
np.random.seed(0) # for reproducibility
numperms=1000 # set to 10000 for more accurate results
all_loadings=np.zeros(numperms)
# permute data
for iperm in tqdm(range(numperms)):
    data_rescaled_perm=pd.DataFrame()
    data_rescaled_perm['HPT'] = data_rescaled[:,0]
    data_rescaled_perm['CPT'] = data_rescaled[:,1]
    data_rescaled_perm['MPT_log_geom'] = np.random.permutation(
                                            data_rescaled[:,2])
```

```
    data_rescaled_perm
    pca = PCA(n_components=3)
    pca.fit(data_rescaled_perm)
    loadings = pca.components_.T * np.sqrt(pca.explained_variance_)
    # contribution of the permuted MPT to PC1
    all_loadings[iperm] = loadings[2,0]

# calculate p-value:
orignal_loading_mpt=original_loadings[2,0]
print("p = ", str(np.sum(all_loadings > orignal_loading_mpt)/len(all_loadings)))
```

```
100%|      | 1000/1000 [00:03<00:00, 268.96it/s]

p =  0.05
```

6.3.2 Results

The permutation-based p-value of the PCA is p=0.049 (with 10000 permutations), providing evidence for a **shared component across all three modalities**.

6.3.3 Interpretation

The most plausible interpretation of these results is that there is a "thermal component" shared between HPT and CPT and another "general component" which is shared across all three modalities.

While in our paper, the latter component is of interest, the mean composite pain sensitivity, as defined by Zunhammer et al., 2016, most probably captures both the "thermal" and the "general" components, thereby meaning a potential bias in the proposed prediction approach (RPN-signature).

To characterize the above mentioned bias, below we evaluate, whether the composite pain sensitivity score of (Zunhammer et al., 2016) and the score predicted by the RPN-signature is correlated with all three modalities.

7 Question Q2. Do the the composite pain sensitivity score of (Zunhammer et al., 2016), similarly to PC1, capture the identified modality-independent component?

7.0.1 Step 1. PC1 vs. the "mean pain sensitivity score" by (Zunhammer et al., 2016)

First we test, to what extent the the "mean pain sensitivity score" by (Zunhammer et al., 2016) (used in our study as prediction target) correlates to PC1 (defined above).

** Possible outcomes and interpretations:** - Due to the significant collinearity (Q1, step 2) and the significant internal consistency (Q1, step 1) of the pain threshold measures, PC1 is most probably very strongly correlated with the mean allowing for using the mean and PC1 interchangeably, and as prediction target in our study.

[23]:
```
# we define a function for computing p-values with permutation test,
# as this will be applied multiple times
def permtest(A, B, nameA="A", nameB="B", numperm=10000):
    cor=np.corrcoef(A, B)[0,1]
    print("cor(" + nameA + "," + nameB + ") = " + str(cor) )
    p_value = permutation_test(A, B,
                               method='approximate',
                               func=lambda x, y: np.sign(cor) *
                                                np.corrcoef(x, y)[1][0],
                               num_rounds=numperm,
                               seed=0)
    if p_value==0:
        print("Permuation-based p < " + str(1.0/numperm))
    else:
        print("Permuation-based p = " + str(p_value))

permtest(df['mean_QST_pain_sensitivity'],
         principal_components[:,0],
         "Zunhammer-score", "PC1")
```

```
cor(Zunhammer-score,PC1) = 0.9259546424719092
Permuation-based p < 0.0001
```

7.0.2 Result

As expected, the mean strongly correlates (R=0.93, p<<0.001) with the first principal component, therefore, most probably, captures the shared component of interest.

Below, we plot the pain thresholds, together with PC1 and the mean pain sensitivity score by (Zunhammer et al., 2016) as a network:

[30]:
```
# Calculate the correlation between individuals.
# We have to transpose first, because the corr function
# calculates the pairwise correlations between columns.
tmpdf=df[["HPT", "CPT", "MPT_log_geom", "mean_QST_pain_sensitivity"]]
tmpdf['PC1']=principal_components[:,0]
# just to fix order of columns
tmpdf=tmpdf[["HPT", "CPT", "MPT_log_geom", "mean_QST_pain_sensitivity", 'PC1']]
corr = tmpdf.corr()
corr = corr.where(np.triu(np.ones(corr.shape)).astype(np.bool))

# Transform it in a links data frame (3 columns only):
links = corr.stack().reset_index()
```

```python
links.columns = ['var1', 'var2','value']
links['value']=np.round(links['value'], decimals=2)

# Remove self correlation (cor(A,A)=1)
links_filtered=links.loc[ (links['var1'] != links['var2']) ]

# Build your graph
G=nx.from_pandas_dataframe(links_filtered, 'var1', 'var2', edge_attr='value')

significant = [(u, v) for (u, v, d)
    in G.edges(data=True) if np.abs(d['value']) > 0.15]
significant_value = [d['value'] for (u, v, d)
    in G.edges(data=True) if np.abs(d['value']) > 0.15]
nonsignificant = [(u, v) for (u, v, d)
    in G.edges(data=True) if np.abs(d['value']) <= 0.15]
nonsignificant_value = [d['value'] for (u, v, d)
    in G.edges(data=True) if np.abs(d['value']) <= 0.15]

# Plot the network:
pos = nx.circular_layout(G)
plt.figure(3,figsize=(12,10))
nx.draw(G, pos, with_labels=True, node_color=['red',
                                              'lightgray',
                                              'lightgray',
                                              'lightgray',
                                              'red',],
        node_size=4000, edge_color='lightgray',
        linewidths=10, width=10, style='dotted')
edges=nx.draw_networkx_edges(G, pos,
                             edge_color=significant_value,
                             edgelist=significant, width=10,
                             edge_cmap=plt.cm.bwr, edge_vmin=-1, edge_vmax=1)

plot=nx.draw_networkx_edge_labels(G,
                             pos,
                             edge_labels=nx.get_edge_attributes(G,'value'),
                             font_color='black',
                             font_size=25)

colorbar=plt.colorbar(edges)
```

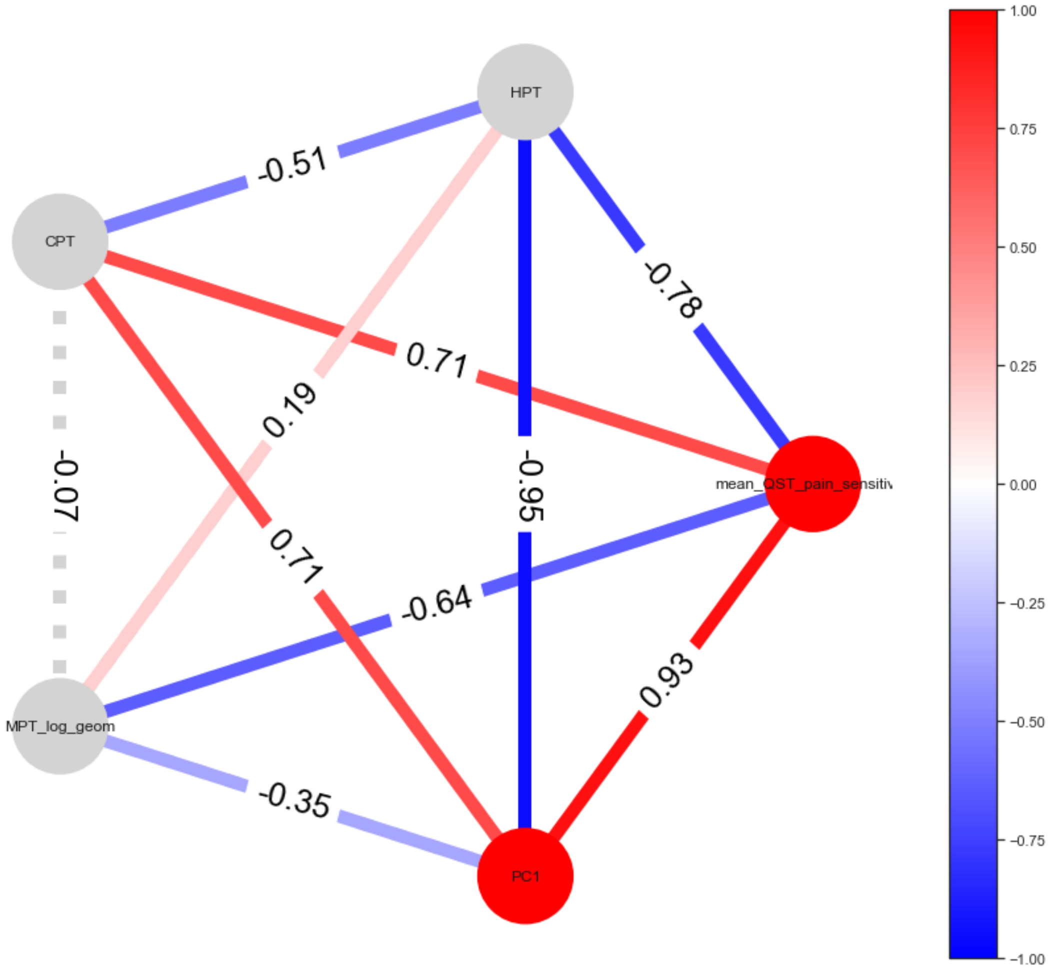

7.0.3 *Supplementary Figure 9. Network representation of the correlation structure across CPT, HPT, MPT, PC1 and the composite score of (Zunhammer et al., 2016).*

Dotted lines and light gray color mean no significance. Solid lines mean significant correlation and are color-coded, according to the legend.

7.0.4 Interpretation

As expected, the composite pain sensitivity scores (red nodes), namely PC1 and the the mean pain sensitivity score by (Zunhammer et al., 2016) (denoted as "mean_QST_pain_sensitivity" on the figure) are *significantly correlated with all modalities*, including MPT. This suggests, that similarly to PC1 (as shown above) the composite pain sensitivity score by (Zunhammer et al., 2016) is also able to capture the shared component of interest.

Below we look at the single data points to confirm that correlations between the composite pain sensitivity score by (Zunhammer et al., 2016) and the single modalities are **not** driven by e.g. outliers.

[31]:
```
# Plot correlations
fig, (ax1, ax2, ax3) = plt.subplots(ncols=3, sharey=True, figsize=(15,5))
sns.regplot(x=df['HPT'], y=df['mean_QST_pain_sensitivity'], ax=ax1)
ax1.set_ylabel('pain sensitivity (Zunhammer)')
sns.regplot(x=df['CPT'], y=df['mean_QST_pain_sensitivity'], ax=ax2)
ax2.set_ylabel('pain sensitivity (Zunhammer)')
sns.regplot(x=df['MPT_log_geom'], y=df['mean_QST_pain_sensitivity'], ax=ax3)
ax3.set_ylabel('pain sensitivity (Zunhammer)')
ran=np.max(df['mean_QST_pain_sensitivity'])-np.
 ↪min(df['mean_QST_pain_sensitivity'])
plt.ylim(np.min(df['mean_QST_pain_sensitivity'])-0.1*ran,
         np.max(df['mean_QST_pain_sensitivity'])+0.1*ran)
ran=np.max(df['MPT_log_geom'])-np.min(df['MPT_log_geom'])
ax3.set_xlim(np.min(df['MPT_log_geom'])-0.1*ran,np.max(df['MPT_log_geom'])+0.
 ↪1*ran)

# compute p-values with permutation test
print("Correlation with the pain sensitivity score of (Zunhammer et al., 2016):
 ↪")
permtest(df['HPT'], df['mean_QST_pain_sensitivity'],
         'HPT', 'pain sensitivity (Zunhammer)')
permtest(df['CPT'], df['mean_QST_pain_sensitivity'],
         'CPT', 'pain sensitivity (Zunhammer)')
permtest(df['MPT_log_geom'], df['mean_QST_pain_sensitivity'],
         'MPT', 'pain sensitivity (Zunhammer)')
```

```
Correlation with the pain sensitivity score of (Zunhammer et al., 2016):
cor(HPT,pain sensitivity (Zunhammer)) = -0.7797640399729854
Permuation-based p < 0.0001
cor(CPT,pain sensitivity (Zunhammer)) = 0.7055498458988412
Permuation-based p < 0.0001
cor(MPT,pain sensitivity (Zunhammer)) = -0.6396260726198744
Permuation-based p < 0.0001
```

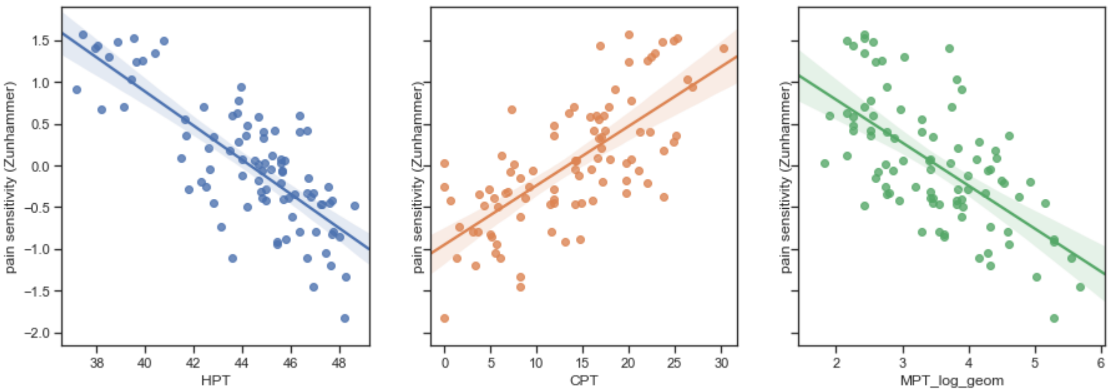

7.0.5 *Supplementary Figure 10. Correlations of CPT, HPT and MPT with the composite pain sensitivity score of Zunhammer et al.*

Units are °C for CPT and HPT and log(mN) for MPT. The correlations of the composite pain sensitivity score with all three modalities were significant (R=-0.78, 0.71 and -0.64, for HPT, CPT and MPT, respectively. p<0.0001 for all correlations).

8 Question Q3. Do we have evidence that the prediction (the RPN-score, trained using the "mean pain sensitivity score" by (Zunhammer et al., 2016)) captures the identified shared, modality-independent component?

So far, we have shown that: - the all three modalities (HPT, CPT and MPT) significantly contribute to the first principal component (PC1) of the data, pointing to the existence of a shared, modality-independent component of pain sensitivity. - the "mean pain sensitivity score" by (Zunhammer et al., 2016), used in our study as a proxy for modality-independent pain sensitivity and prediction target, very strongly correlates with PC1, therefore, most probably incorporates the shared, modality-independent component of interest

** *NOTE:* ** The RPN-signature was trained with the the "mean pain sensitivity score" by (Zunhammer et al., 2016) and was "blind" to the single scores, already during the training procedure.

At this point, however, we cannot exclude the possibility, that the the composite pain sensitivity score by (Zunhammer et al., 2016) is dominated by components other than the modality-independent component of interest (e.g. thermal modality-specific or noise) so such a great extent that using it as a prediction target ends up in loosing the "modality-independency" and remains predictive to only one or two modalities.

This would make the RPN-signature unstable for adding or removing a sensory modality from the composite score.

We test, how well the *composite score-based* RPN prediction generalizes to the single modalities by leaving out modalities when computing the observed composite score. Specifically, this involves an analysis of leaving out one modality (Step 1) and leaving out two modalities, i.e. retaining only one modality.

8.0.1 Step 1. "Leave-one-modality-out" analysis

In this analysis, we calculate two-modality summary scores with the same method used for the Zunhammer-score. Comparing these "leave-one-modality-out" scores to the RPN-score provides insights into the generalizability of the RPN-signature to combinations of different modalities and potentially, adding a new modality.

[32]:
```
# calculate leave-one-modality-out summary scores
# (analogously to the method used by Zunhammer et al.)
# means and standard deviations from Study1 (Bochum-sample):
# these values are hard-coded into the
# calculation of the score of (Zunhammer et al., 2016),
# so that its calculation is independent of the data at hand
b_hpt_mean=44.21297
b_hpt_sd=2.799718
b_cpt_mean=14.35385
b_cpt_sd=7.595601
b_mpt_mean=3.617695
b_mpt_sd=0.801178

hpt_scaled=-(df['HPT']-b_hpt_mean)/b_hpt_sd
cpt_scaled=(df['CPT']-b_cpt_mean)/b_cpt_sd
mpt_scaled=-(df['MPT_log_geom']-b_mpt_mean)/b_mpt_sd

df_lomo=df #lomo:leave-one-modality-out
df['HPT_CPT']=(hpt_scaled+cpt_scaled)/2
df['HPT_MPT']=(hpt_scaled+mpt_scaled)/2
df['CPT_MPT']=(cpt_scaled+mpt_scaled)/2

# Plot correlation with the "RPN-score"
fig, (ax1, ax2, ax3) = plt.subplots(ncols=3, sharey=True, figsize=(15,5))
sns.regplot(x=df['HPT_CPT'], y=df['prediction'], ax=ax1)
sns.regplot(x=df['HPT_MPT'], y=df['prediction'], ax=ax2)
sns.regplot(x=df['CPT_MPT'], y=df['prediction'], ax=ax3)
ax1.set_ylabel('RPN-score')
ax2.set_ylabel('RPN-score')
ax3.set_ylabel('RPN-score')

# compute p-values with permutation test
print("Correlation with the RPN-score:")
permtest(df['HPT_CPT'], df['prediction'], 'composite(HPT,CPT)', 'RPN-score')
permtest(df['HPT_MPT'], df['prediction'], 'composite(HPT,MPT)', 'RPN-score')
```

```
permtest(df['CPT_MPT'], df['prediction'], 'composite(CPT,MPT)', 'RPN-score')
```

```
Correlation with the RPN-score:
cor(composite(HPT,CPT),RPN-score) = 0.4575562457450685
Permuation-based p < 0.0001
cor(composite(HPT,MPT),RPN-score) = 0.4291506440039318
Permuation-based p < 0.0001
cor(composite(CPT,MPT),RPN-score) = 0.40012762722771095
Permuation-based p < 0.0001
```

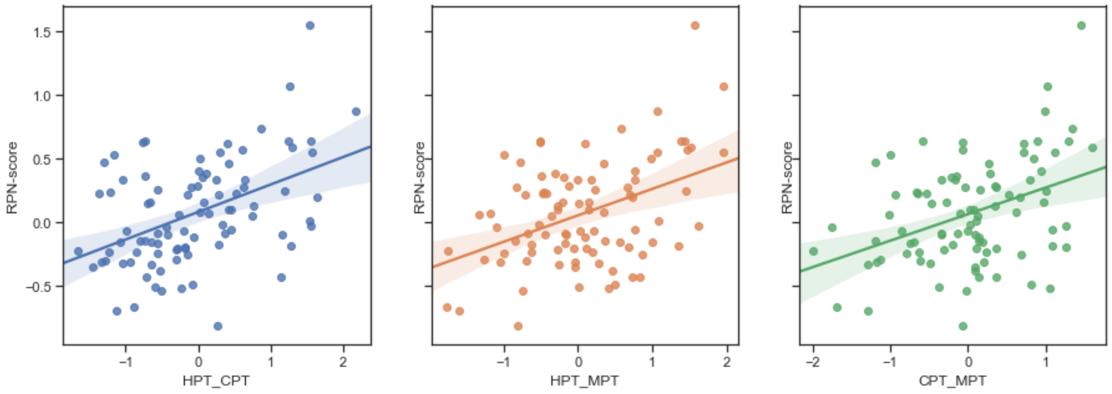

8.0.2 *Supplementary Figure 11. Correlations of the "leave-one-modality-out" composite variables with the predicted pain sensitivity score (the RPN-score).*

Units are arbitrary, based on the standardized variables. The correlations of the RPN-score with all three "leave-one-modality-out" composite variables were significant (R=0.46, 0.43 and 0.40, for the "leave-one-modality-out" composite variables HPT-CPT, HPT-MPT and CPT-MPT, respectively. p<0.0001 for all correlations).

8.0.3 Result

All possible leave-one-modality-out scores were significantly predicted by the RPN-score.

8.0.4 Interpretation

The RPN-signature is **not** driven by one modality only, and stays robust for different definitions of "composite pain sensitivity".

8.0.5 Step 2. "Leave-two-modalities-out" analysis

Leaving out two modalities equals with simply testing the relationship of the composite RPN-score with the single modalities (HPT, CPT and MPT)

[33]:
```
# Plot correlation with the "RPN-score"
fig, (ax1, ax2, ax3) = plt.subplots(ncols=3, sharey=True, figsize=(15,5))
sns.regplot(x=df['HPT'], y=df['prediction'], ax=ax1)
sns.regplot(x=df['CPT'], y=df['prediction'], ax=ax2)
sns.regplot(x=df['MPT_log_geom'], y=df['prediction'], ax=ax3)
ran=np.max(df['prediction'])-np.min(df['prediction'])
plt.ylim(np.min(df['prediction'])-0.1*ran, np.max(df['prediction'])+0.1*ran)
ran=np.max(df['MPT_log_geom'])-np.min(df['MPT_log_geom'])
ax3.set_xlim(np.min(df['MPT_log_geom'])-0.1*ran,np.max(df['MPT_log_geom'])+0.
↪1*ran)
ax1.set_ylabel('RPN-score')
ax2.set_ylabel('RPN-score')
ax3.set_ylabel('RPN-score')

# compute p-values with permutation test
print("Correlation with the RPN-score:")
permtest(df['HPT'], df['prediction'], 'HPT', 'RPN-score')
permtest(df['CPT'], df['prediction'], 'CPT', 'RPN-score')
permtest(df['MPT_log_geom'], df['prediction'], 'MPT', 'RPN-score')
```

```
Correlation with the RPN-score:
cor(HPT,RPN-score) = -0.43644372163377854
Permuation-based p < 0.0001
cor(CPT,RPN-score) = 0.35617457477513276
Permuation-based p = 0.0005
cor(MPT,RPN-score) = -0.24024492612674364
Permuation-based p = 0.0129
```

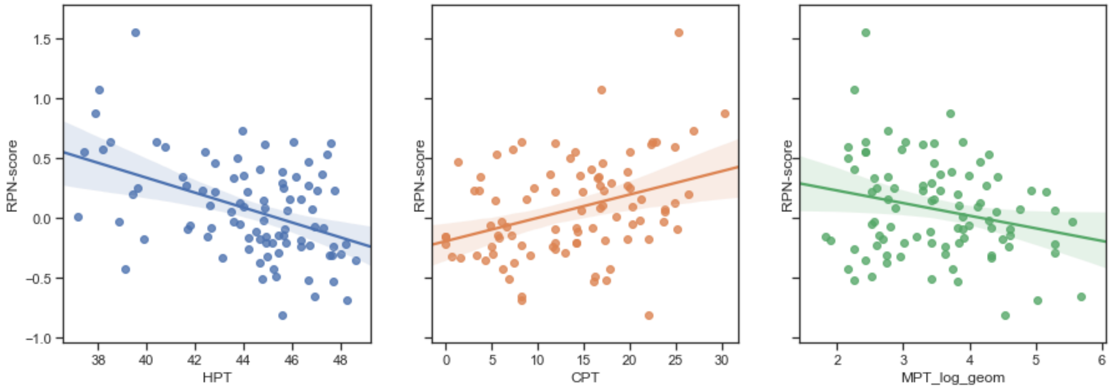

8.0.6 *Supplementary Figure 12. Correlations of the "leave-two-modality-out" composite variables with the predicted pain sensitivity score (the RPN-score).*

The correlations of the RPN-score with all three "leave-two-modality-out" composite variables were significant (R=-0.44, 0.36 and -0.24, for the "leave-two-modality-out" composite variables, i.e. the single pain thresholds HPT, CPT and MPT, respectively. p<0.05 for all correlations).

8.0.7 Result

All possible "leave-two-modality-out" scores, i.e. all three pain modalities were significantly predicted by RPN-score.

8.0.8 Interpretation

The RPN-signature is **not** exclusively driven by any combination of two modalities, specifically, not only driven by the thermal thresholds. The RPN-score holds predictive value for all modalities, even though it was specifically trained to predict the modality-independent composite pain sensitivity score by (Zunhammer et al., 2016).

8.0.9 *Figure SA1.7. Correlations of the "leave-two-modality-out" variables, i.e. the single modalities with the predicted pain sensitivity score (the RPN-score).*

Units are °C for CPT and HPT and log(mN) for MPT. The correlations of the RPN-score with all three modalities were significant (R=-0.44, 0.36 and -0.24, for HPT, CPT and MPT, respectively. P-values: pHPT,RPN < 0.0001, pCPT,RPN=0.0005, pMPT,RPN=0.01.

9 Question Q4. To what extent is the RPN-score biased towards any of the modalities? Is its correlation with the single thresholds lower than excpeted from its correlation to the Zunhammer-score?

9.0.1 Step 1. Analyis-of-bias

This analysis aims to exclude the possibility that the RPN-score is biased e.g. towards thermal thresholds.

We will employ "simulated" RPN-scores that are constructed by adding orthogonalized Gaussian noise to the pain sensitivity score of (Zunhammer et al, 2016), so that the correlation of these simulated scores with the original pain sensitivity score equals to its correlation to the RPN-score. For the sake of simplicity, we refer to these artificially generated scores as "simulated RPN-scores".

The correlation of many simulated RPN-scores to the single pain thresholds (HPT, CPT, MPT) will be used to construct a null distribution for these correlations. Then, the actual "RPN-score

vs. single threshold" correlations will be contrasted to these null distributions to obtain p-values. The test is one sided, testing for "negative bias" (smaller correlation than expected from the null).

Possible outcomes and interpretations: - a significant p-value implies a strong bias against the given modality, meaning that the degree to which the RPN-signature generalizes to the single pain thresholds significantly differs from what we can expect given its correlation to the pain sensitivity score by Zunhammer et al. - p-values greater than the alpha-level (p>0.05) means that our data provides *no evidence* for significant bias of the RPN-score towards any of the single pain modalities.

[41]:
```
old_df=df
df=df#study3.dropna()

cor_Zunhammer_rpn = np.corrcoef(df['mean_QST_pain_sensitivity'],
                                df['prediction'])[0,1]

np.random.seed(1)
num_sim=1000

# a nice tricky function to genberate random simulated RPN scores:
# variables with a given correlation to the composite score of Zunhammer et al.
# ported to python from:
# https://stats.stackexchange.com/questions/15011/
 ↪generate-a-random-variable-with-a-defined-correlation-to-an-existing-variables
def get_simulated_RPN(rho=cor_Zunhammer_rpn,
                      y=df['mean_QST_pain_sensitivity'],
                      x=None,
                      seed=None):
    if (not x):
        if seed is not None:
            np.random.seed(seed)
        x = np.random.normal(0,1,len(y))
    y=np.array(y)
    x=np.array(x)
    y_perp = sm.OLS(x,sm.tools.tools.add_constant(y)).fit().resid
    return( rho * np.std(y_perp, ddof=1) * y +
            y_perp * np.std(y, ddof=1) * np.sqrt(1 - rho**2.0) )

rhos_simRPN_Zunhammer=np.zeros(num_sim)
rhos_simRPN_HPT=np.zeros(num_sim)
rhos_simRPN_CPT=np.zeros(num_sim)
rhos_simRPN_MPT=np.zeros(num_sim)

Zunhammer_std = (df['mean_QST_pain_sensitivity']-
    np.mean(df['mean_QST_pain_sensitivity']))/-np.std(
                                        df['mean_QST_pain_sensitivity'])

# simulate simulated RPN scores
for sim_i in tqdm(range(num_sim)):
```

```
    # cereate a "noisy Zunhammer"
    simRPN=get_simulated_RPN()
    # calculate the correlation of the simulated RPN scores and
    # the observed composite score by Zunhammer et al.
    rhos_simRPN_Zunhammer[sim_i]=np.corrcoef(df['mean_QST_pain_sensitivity'],
                                              simRPN)[0,1]
    # calculate the correlation between the
    # simulated RPN scores and the single pain thershodls
    rhos_simRPN_HPT[sim_i]=np.corrcoef(df['HPT'], simRPN)[0,1]
    rhos_simRPN_CPT[sim_i]=np.corrcoef(df['CPT'], simRPN)[0,1]
    rhos_simRPN_MPT[sim_i]=np.corrcoef(df['MPT_log_geom'], simRPN)[0,1]


# assess p-values based on the null distributions of correlations
# between the simulated RPN scores and HPT
cor_HPT_RPN=np.corrcoef(df['HPT'], df['prediction'])[0,1]
hist=plt.hist(rhos_simRPN_HPT)
plt.title(
    "Histogram of the correlations between HPT and the \"simulated␣
 ↪RPN-scores\"")
plt.xlabel("Correlation coefficient")
plt.axvline(cor_HPT_RPN, color='k', linestyle='dashed', linewidth=2)
text=plt.text(cor_HPT_RPN, np.max(hist[0])*0.9,
    " cor(HPT, RPN)=" + str(np.round(cor_HPT_RPN, 2)))
plt.show()
print("p = ", str(np.sum(rhos_simRPN_HPT > cor_HPT_RPN)/num_sim) )


#assess p-values based on the null distributions of
# correlations between the simulated RPN scores and CPT
cor_CPT_RPN=np.corrcoef(df['CPT'], df['prediction'])[0,1]
hist=plt.hist(rhos_simRPN_CPT)
plt.title(
    "Histogram of the correlations between CPT and the \"simulated␣
 ↪RPN-scores\"")
plt.xlabel("Correlation coefficient")
plt.axvline(cor_CPT_RPN, color='k', linestyle='dashed', linewidth=2)
text=plt.text(cor_CPT_RPN, np.max(hist[0])*0.9,
    " cor(CPT, RPN)=" + str(np.round(cor_CPT_RPN, 2)))
plt.show()
print("p = ", str(np.sum(rhos_simRPN_CPT < cor_CPT_RPN)/num_sim) )


# assess p-values based on the null distributions of
# correlations between the simulated RPN scores and MPT
cor_MPT_RPN=np.corrcoef(df['MPT_log_geom'], df['prediction'])[0,1]
hist=plt.hist(rhos_simRPN_MPT)
```

```
plt.title(
    "Histogram of the correlations between MPT and the \"simulated␣
 ↪RPN-scores\"")
plt.xlabel("Correlation coefficient")
plt.axvline(cor_MPT_RPN, color='k', linestyle='dashed', linewidth=2)
text=plt.text(cor_MPT_RPN, np.max(hist[0])*0.9,
    " cor(MPT, RPN)=" + str(np.round(cor_MPT_RPN, 2)))
plt.show()
print("p = ", str(np.sum(rhos_simRPN_MPT > cor_MPT_RPN)/num_sim) )

print("Correlation of the Composite score of Zunhammer et al. and the RPN-score:
 ↪ "
    + str(cor_Zunhammer_rpn))

df=old_df
```

```
100%|     | 1000/1000 [00:01<00:00, 784.92it/s]
```

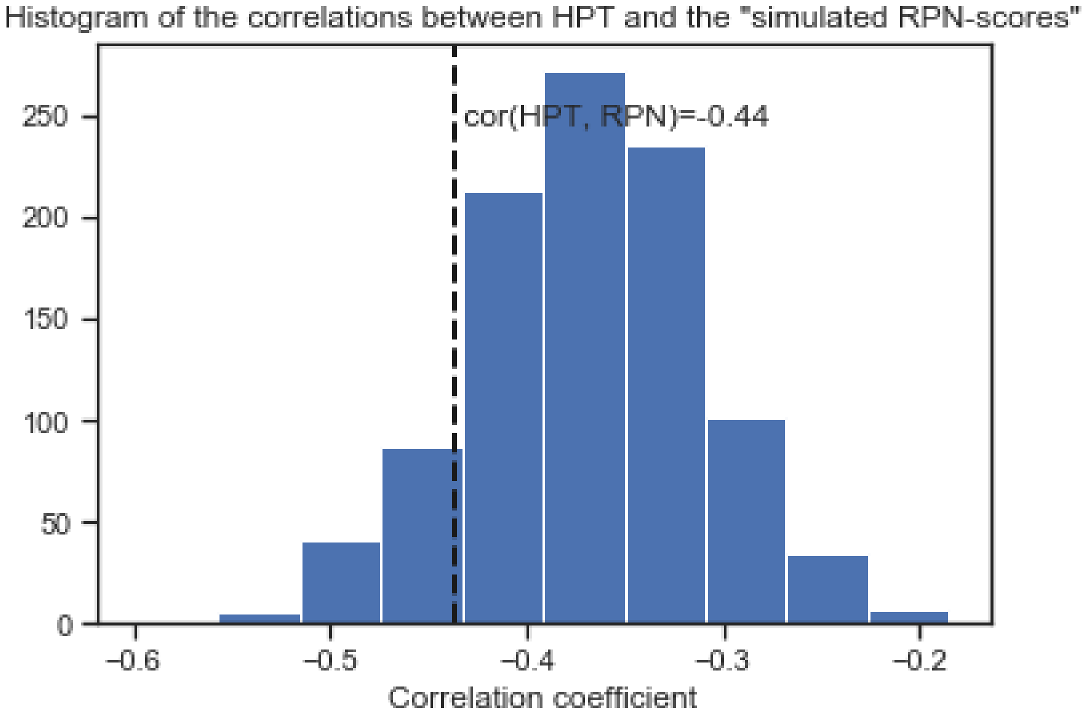


```
p =  0.873
```

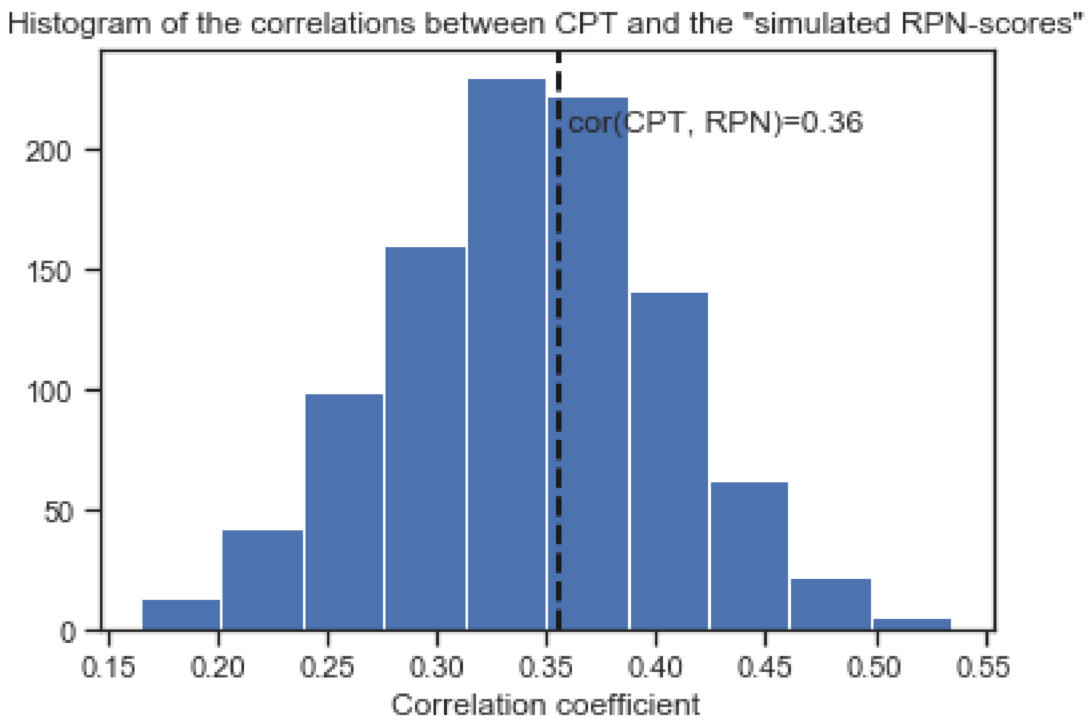
Histogram of the correlations between CPT and the "simulated RPN-scores"
cor(CPT, RPN)=0.36
0
50
100
150
200
0.15
0.20
0.25
0.30
0.35
0.40
0.45
0.50
0.55
Correlation coefficient

p = 0.583

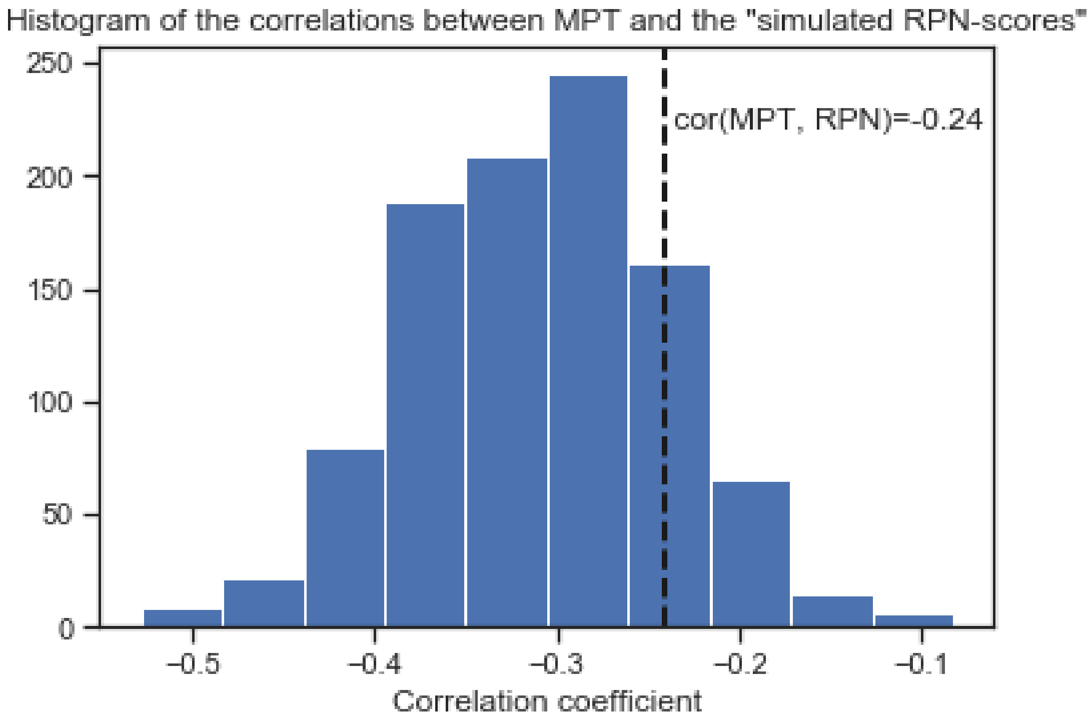
Histogram of the correlations between MPT and the "simulated RPN-scores"
cor(MPT, RPN)=-0.24
0
50
100
150
200
250
−0.5
−0.4
−0.3
−0.2
−0.1
Correlation coefficient

```
p =  0.166
Correlation of the Composite score of Zunhammer et al. and the RPN-score:
0.4801750952950685
```

9.0.2 *Supplementary Figure 13. Analysis-of-bias.*

Histograms shown the null distributions of the correlations of simulated predictive scores with HPT, CPT and MPT, respectively. Dashed lines imply the actual correlation of the given pain threshold with the true RPN-score. P-values are above 0.05 for all three sensory modalities, showing no evidence of negative bias of the RPN-score in any of the modalities.

9.0.3 Results

All p-values are gerater than 0.05.

9.0.4 Interpretation

We found no evidence of negative bias towards any of the sensory modalities.

10 Conclusion

In this python notebook, we have performed a multi-stage analysis to investigate the rationale for using the composite pain sensitivity score as defined by (Zunhammer et al., 2016) for predictive modelling purposes.

We have found that: - (Q1) There **is** a common, modality-independent component shared across the investigated pain modalities. - (Q2) The the composite score of (Zunhammer et al., 2016) does capture this shared, modality-independent component. - (Q3) The prediction of the RPN-signature (the RPN-score) also captures the identified modality-independent component of pain sensitivity. - (Q4) The RPN-score was not found to be significantly biased towards/against any of the modalities.

We conclude, that the RPN-score represents a modality independent component of pain sensitivity.

11 Supplementary References

(Babbie, 2016) Babbie ER. The basics of social research. Cengage learning; page 158. 2013.

(Bobko et al, 2007) Bobko P, Roth PL, Buster MA. The usefulness of unit weights in creating composite scores: A literature review, application to content validity, and meta-analysis. Organizational Research Methods. 2007 Oct;10(4):689-709.

(Dubin & Patapoutian, 2010) Dubin AE, Patapoutian A. Nociceptors: the sensors of the pain pathway. The Journal of clinical investigation. 2010 Nov 1;120(11):3760-72.

(Dunn et al., 2014) Dunn TJ, Baguley T, Brunsden V. From alpha to omega: A practical solution to the pervasive problem of internal consistency estimation. British journal of psychology. 2014 Aug;105(3):399-412.

(Haugen et al., 2010) Haugen IK, Slatkowsky-Christensen B, Lessem J, Kvien TK. The responsiveness of joint counts, patient-reported measures and proposed composite scores in hand osteoarthritis: analyses from a placebo-controlled trial. Annals of the rheumatic diseases. 2010 Aug 1;69(8):1436-40.

(Jensen et al., 1999) Jensen MP, Turner JA, Romano JM, Fisher LD. Comparative reliability and validity of chronic pain intensity measures. Pain. 1999 Nov 1;83(2):157-62.

(Jensen et al., 2002) Jensen MP, Chen C, Brugger AM. Postsurgical pain outcome assessment. Pain. 2002 Sep 1;99(1-2):101-9.

(O'Neill et al., 2014) O'Neill S, Manniche C, Graven-Nielsen T, Arendt-Nielsen L. Association between a composite score of pain sensitivity and clinical parameters in low-back pain. The Clinical journal of pain. 2014 Oct 1;30(10):831-8.

(Rolke et al., 2006) Rolke R, Baron R, Maier CA, Tölle TR, Treede RD, Beyer A, Binder A, Birbaumer N, Birklein F, Bötefür IC, Braune S. Quantitative sensory testing in the German Research Network on Neuropathic Pain (DFNS): standardized protocol and reference values. Pain. 2006 Aug 1;123(3):231-43.

(Song et al., 2013) Song MK, Lin FC, Ward SE, Fine JP. Composite variables: when and how. Nursing research. 2013 Jan;62(1):45.

(Starkweather et al., 2016) Starkweather AR, Heineman A, Storey S, Rubia G, Lyon DE, Greenspan J, Dorsey SG. Methods to measure peripheral and central sensitization using quantitative sensory testing: A focus on individuals with low back pain. Applied Nursing Research. 2016 Feb 1;29:237-41.

(Victor et al., 2008) Victor TW, Jensen MP, Gammaitoni AR, Gould EM, White RE, Galer BS. The dimensions of pain quality: factor analysis of the Pain Quality Assessment Scale. The Clinical journal of pain. 2008 Jul 1;24(6):550-5.

(Zinbarg et al., 2006) Zinbarg RE, Yovel I, Revelle W, McDonald RP. Estimating generalizability to a latent variable common to all of a scale's indicators: A comparison of estimators for h. Applied Psychological Measurement. 2006 Mar;30(2):121-44.

(Zunhammer et al., 2016) Zunhammer M, Schweizer LM, Witte V, Harris RE, Bingel U, Schmidt-Wilcke T. Combined glutamate and glutamine levels in pain-processing brain regions are associated with individual pain sensitivity. Pain. 2016 Oct 1;157(10):2248-56.

Supplementary Note 2

November 19, 2019

1 Analysis of the stability of the RPN-signature to parcellation-related effects.

1.1 *Supplementary Analysis 2 for the paper "Pain-free resting-state functional brain connectivity predicts individual pain sensitivity"*

Supplementary Analyses 2 is also available at the following link:

https://raw.githack.com/spisakt/RPN-signature/master/notebooks/Supplementary_Analysis_2.html

Alternatively, to edit and run the code:

https://nbviewer.jupyter.org/github/pni-lab/RPN-signature/blob/master/notebooks/Supplementary_Analysis_2
>By clicking on "Execute on Binder" in the top right corner, the reviewers can enter the interactive mode where the code of the analysis can be edited and run in a dedicated python environment.

2 Stability of the RPN-signature to the regions-of-interest system

Initially, we import the required python modules and load the data (composite pain sensitivity, RPN-score, regional timeseries).

[7]:
```
# import neccessary modules and load all data
import pandas as pd
import numpy as np
import matplotlib.pyplot as plt
import seaborn as sns; sns.set()
sns.set(style="ticks")
import warnings
warnings.filterwarnings('ignore')
from sklearn.preprocessing import StandardScaler
from sklearn.externals import joblib
from tqdm import tqdm as tqdm_base
# just a hack for a tqdm progress bar issue in python 2 notebooks
# https://github.com/tqdm/tqdm/issues/375
def tqdm(*args, **kwargs):
    if hasattr(tqdm_base, '_instances'):
        for instance in list(tqdm_base._instances):
```

```
            tqdm_base._decr_instances(instance)
    return tqdm_base(*args, **kwargs)

# Load all data:
study1 = pd.read_csv("../res/bochum_sample_excl.csv")[["HPT",
                                                        "CPT",
                                                        "MPT_log_geom",
                                                       ␣
 ↪"mean_QST_pain_sensitivity",
                                                        "prediction"]]
study2 = pd.read_csv("../res/essen_sample_excl.csv")[["HPT",
                                                       "CPT",
                                                       "MPT_log_geom",
                                                      ␣
 ↪"mean_QST_pain_sensitivity",
                                                       "prediction"]]
study3 = pd.read_csv("../res/szeged_sample_excl.csv")[["HPT",
                                                        "CPT",
                                                        "MPT_log_geom",
                                                       ␣
 ↪"mean_QST_pain_sensitivity",
                                                        "prediction"]]
# merge datasets
df = pd.concat([study1, study2, study3])

# load the trained RPN-model
rpn = joblib.load("../res/predictive_model.sav")

# load features for each study
X_bochum = joblib.load("../res/feature_bochum.sav")
X_essen = joblib.load("../res/feature_essen.sav")
X_szeged = joblib.load("../res/feature_szeged.sav")

X=np.concatenate((X_bochum, X_essen, X_szeged))
```

2.1 Step 1. Stability to the definition region-boundaries (e.g. mixed signal)

Next to full drop-out of some of the regions another issue on the level of the regional timeseries can be a decreased signal-to-noise ratio. Such an effect can be caused, for instance, by a generally low measurement quality or a less accurate definition of regions (e.g. co-registration inaccuracies or use of another brain atlas).

First, we define a function to add a given proportion of Gaussian noise to *all* of the regional timeseries. >new_timeseries = original_timeseries + noise_weight * Guassian_random(mean(original_timeseries), sd(original_timeseries))

- noise_weight = 0 means no additional noise at all,

- noise_weight = 1 means an equal amount of original signal and additional noise

[9]:
```
def mix_region(mixing_weight=0):
    X_noisy=X.copy()
    scaler = StandardScaler()
    scaler.fit(X_noisy)
    X_noisy = X_noisy + mixing_weight * np.random.normal(scaler.mean_,
                                                          scaler.scale_,
                                                          X_noisy.shape)
    predicted = rpn.predict(X_noisy)

    # calculate a reference value by simply adding noise to the original␣
 ↪prediction
    predicted_orig = rpn.predict(X)
    reference=predicted_orig+mixing_weight *np.random.normal(np.
 ↪mean(predicted_orig),
                                                              np.
 ↪std(predicted_orig),
                                                              predicted_orig.
 ↪shape)

    return(predicted, reference)
```

Next, we investigate how prediction accuracy changes as a function of the amount of noise.

[10]:
```
numiter=100
weights=np.linspace(start=0, stop=4, num=10)
noise_weight=np.zeros(len(weights)*numiter)
correlation=np.zeros(len(weights)*numiter)
correlation_reference=np.zeros(len(weights)*numiter)

np.random.seed(0)
idx=0
for w in tqdm(weights):
    for iter in range(numiter):
        ret = mix_region(w)
        correlation[idx] = np.corrcoef(df['mean_QST_pain_sensitivity'],
                                       ret[0])[0,1]
        correlation_reference[idx] = np.
 ↪corrcoef(df['mean_QST_pain_sensitivity'],
                                                 ret[1])[0,1]
        noise_weight[idx] = w
        idx=idx+1

data=pd.DataFrame({
    'noise_weight' : noise_weight,
    'correlation' : correlation,
```

```
    'reference' : correlation_reference
})
```

```
100%|     | 10/10 [00:45<00:00,  4.54s/it]
```

[11]:
```
# plot mixed-signal effect
ax=sns.lineplot(x="noise_weight", y="correlation", ci=95, data=data)
ax=sns.lineplot(x="noise_weight", y="reference", ci=95, data=data)
ax.axhline(0.2061, ls='--')

plt.show()
```

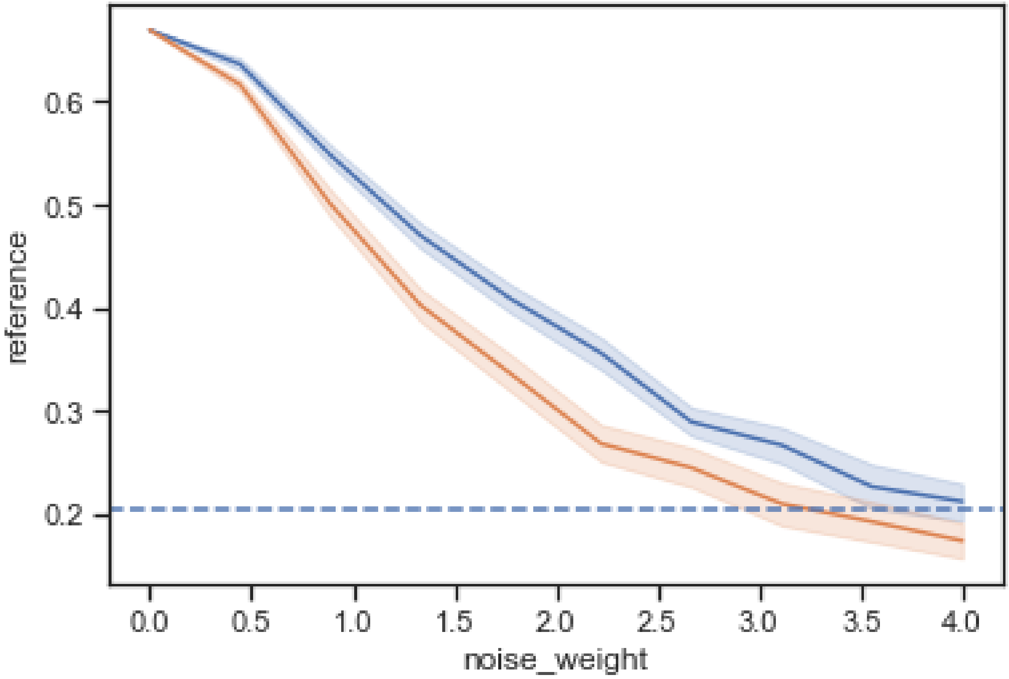

2.1.1 *Supplementary Figure 14. The tolerance of the RPN-signature to noise added to the regional timecourses.*

The mean correlation (and 95% confidence intervals, blue) between predicted and observed pain sensitivity is plotted as a function of the amount of noise added to all timecourses, simultaneously. As a reference, we plot the case of adding the same amount of noise simply to the final prediction (with 95% confidence intervals, orange), i.e. with the regional timeseries unchanged. Dashed line denotes R=0.206, i.e. the correlation value belonging to the p=0.05 significance threshold.

The RPN-signature displays a remarkable robustness to reductions in the signal-to-noise ratios of the regional signals (which can originate e.g. co-registration inaccuracies, BOLD-artefacts or “mixed

signal" due to suboptimal parcellation). The RPN-signature tolerates "mixed signal" effects on the level of regional timeseries significantly better than expected for a single-region-based marker.

2.2 Step 2. Stability to region drop-out

Here, we investigate how robust the RPN-signature is to region drop-out, that is, artificially zeroing out the timecourse of a given number of RPN-regions. On real data, a similar (although not so dramatic) drop-out can be caused e.g. by signal drop-out due to susceptibility artifact (typically in medial frontal regions). We simulate drop out be zeroing out N regions randomly and plot the histogram of correlations of the RPN-scores computed with random drop-out (drop-out score) with the measured composite pain sensitivity.

First, we define a function to perform the "drop-out" of a given number of regions.

[12]:
```
def drop_out_region(num_drop_out=0):
    #load atlas labels
    labels = pd.read_csv("../data/atlas_relabeled.tsv",
                         sep="\t")[["index","labels", "modules"]]
    labels.loc[-1] = [0, "aMEAN_GM", "aMEAN_GM"]  # adding a row
    labels.index = labels.index + 1  # shifting index
    labels = labels.sort_index()  # sorting by index

    # get coefficients of the RPN
    RES = np.zeros(len(labels)*(len(labels)-1)/2)
    featuremask = rpn.named_steps['fsel'].get_support()
    RES[featuremask] = rpn.named_steps['model'].coef_

    # zero-out some regions by randomly selecting from those having a nonzero␣
↪coef
    indices=np.random.choice(np.nonzero(RES)[0], num_drop_out, replace=False)
    X_drop=X.copy()
    X_drop[:, indices]=0

    predicted = rpn.predict(X_drop)
    return(predicted)
```

Next, we investigate how the correlation changes for various drop-out values.

[13]:
```
numiter=50
max_drop_N=22
num_dropped=np.zeros(max_drop_N*numiter)
correlation=np.zeros(max_drop_N*numiter)

np.random.seed(0)
idx=0
for dropN in tqdm(range(max_drop_N)):
    for iter in range(numiter):
```

```
        correlation[idx] = np.corrcoef(df['mean_QST_pain_sensitivity'],
                                       drop_out_region(dropN))[0,1]
        num_dropped[idx]=dropN
        idx=idx+1

data=pd.DataFrame({
    'num_dropped' : num_dropped,
    'correlation' : correlation
})
```

```
100%|      | 22/22 [00:52<00:00,  2.37s/it]
```

[14]:

```
# plot drop-out effect
ax=sns.lineplot(x="num_dropped", y="correlation", ci=95, data=data)
ax.axhline(0.2061, ls='--')
plt.show()
```

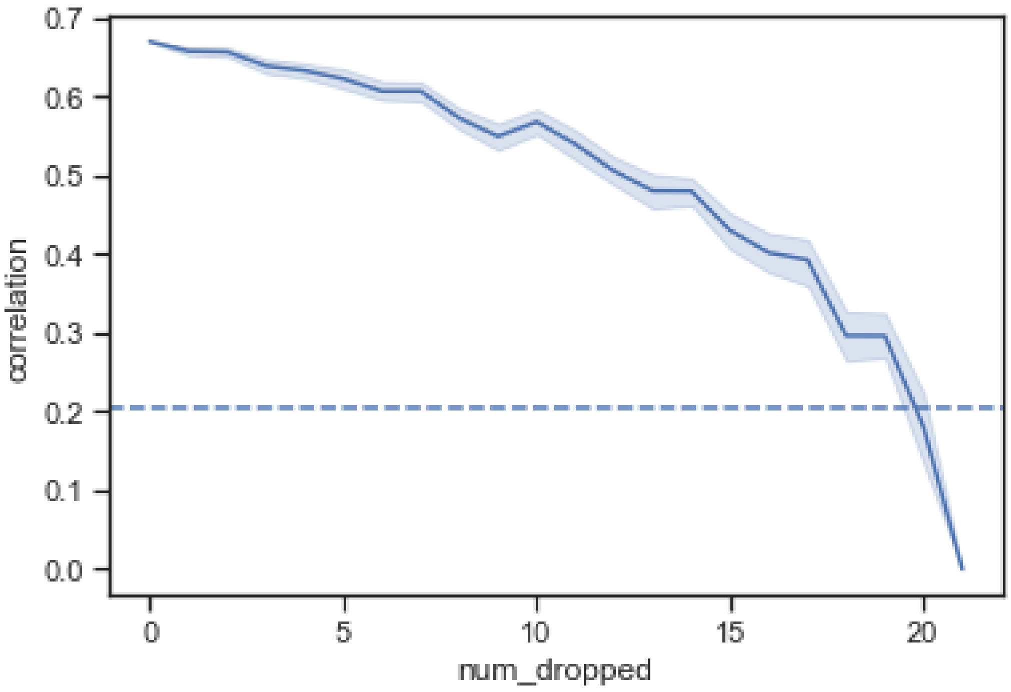

2.2.1 *Supplementary Figure 15. The tolerance of the RPN-signature to region drop-out.*

The mean (and 95% confidence intervals) correlation between predicted and observed pain sensitivity is plotted as a function of the number of regions dropped out randomly by setting their timecourse to constant zero. Dashed line denotes R=0.206, i.e. the correlation value belonging to the p=0.05

significance threshold. The analysis revealed a considerable robustness to drop-out; the average prediction accuracy remained significant by the random drop-out of up to 19 regions out of 21, although – as expected - prediction accuracy constantly decreased with an increasing number of dropped regions.
