# Supplementary material for: Pain-free resting-state functional brain connectivity predicts individual pain sensitivity
Source: Nat Commun. 2020 Jan 10;11:187. doi: 10.1038/s41467-019-13785-z (PMC6954277; doi:10.1038/s41467-019-13785-z)
Supplement: Supplementary file 3 — Reporting Summary [file 41467_2019_13785_MOESM3_ESM.pdf]

nature research

Corresponding author(s): Tamas Spisak

Last updated by author(s): Jun 7, 2019

Reporting Summary

Nature Research wishes to improve the reproducibility of the work that we publish. This form provides structure for consistency and transparency in reporting. For further information on Nature Research policies, see Authors & Referees and the Editorial Policy Checklist.

Statistics

For all statistical analyses, confirm that the following items are present in the figure legend, table legend, main text, or Methods section.

n/a	Confirmed	
☐	☒	The exact sample size (*n*) for each experimental group/condition, given as a discrete number and unit of measurement
☐	☒	A statement on whether measurements were taken from distinct samples or whether the same sample was measured repeatedly
☐	☒	The statistical test(s) used AND whether they are one- or two-sided<br>*Only common tests should be described solely by name; describe more complex techniques in the Methods section.*
☐	☒	A description of all covariates tested
☐	☒	A description of any assumptions or corrections, such as tests of normality and adjustment for multiple comparisons
☐	☒	A full description of the statistical parameters including central tendency (e.g. means) or other basic estimates (e.g. regression coefficient) AND variation (e.g. standard deviation) or associated estimates of uncertainty (e.g. confidence intervals)
☐	☒	For null hypothesis testing, the test statistic (e.g. *F*, *t*, *r*) with confidence intervals, effect sizes, degrees of freedom and *P* value noted<br>*Give P values as exact values whenever suitable.*
☒	☐	For Bayesian analysis, information on the choice of priors and Markov chain Monte Carlo settings
☐	☒	For hierarchical and complex designs, identification of the appropriate level for tests and full reporting of outcomes
☐	☒	Estimates of effect sizes (e.g. Cohen's *d*, Pearson's *r*), indicating how they were calculated

Our web collection on statistics for biologists contains articles on many of the points above.

Software and code

Policy information about availability of computer code

Data collection	MR image acquisition: Siemens Syngo VX57N<br>Recording behavioral data: Medoc Main Station (MMS), Arbel 6.3.7.20
Data analysis	Digitalizing questionnaire data: Microsoft Excel<br>Prerocessing MRI data, training and validating model: "PUMI" in-house software library system (https://github.com/spisakt/PUMI, v0.1.1a), "RPN-signature" in-house predictive modeling pipeline (https://github.com/spisakt/RPN-signature, v0.1.5).<br>The processing pipeline utilizes FSL (v6.0.1), AFNI (AFNI_17.3.03), nipype (v1.1.9), ANTs (v2.2.0.dev62-g074d4), python packages: numpy=1.15.4, scipy=1.1.0, scikit-learn=0.19.1, matplotlib=2.2.2, pandas=0.23.4, libxml2=2.9.8, libxslt=1.1.32, graphviz=2.40.1, traits=4.6.0, statsmodels=0.9.0, bids==0.0 nilearn==0.5.0 seaborn==0.9.0.<br><br>The full analysis pipeline is released as a BIDS-app and conserved into the Docker image tspisak/rpn-signature:v0.1.5 deposited on dockerhub: https://hub.docker.com/r/tspisak/rpn-signature.<br><br>Installation and usage information is available on the project webpage: https://spisakt.github.io/RPN-signature

For manuscripts utilizing custom algorithms or software that are central to the research but not yet described in published literature, software must be made available to editors/reviewers. We strongly encourage code deposition in a community repository (e.g. GitHub). See the Nature Research guidelines for submitting code & software for further information.

Data

Policy information about availability of data

All manuscripts must include a data availability statement. This statement should provide the following information, where applicable:

- Accession codes, unique identifiers, or web links for publicly available datasets
- A list of figures that have associated raw data
- A description of any restrictions on data availability

Processed data (regional timeseries) and source code are deposited at https://github.com/spisakt/RPN-signature. Raw imaging data is available at openneuro.org. The RPN-signature scores can be computed based on structural and resting-state functional datasets by the software tool with the same name. The RPN-signature software tool consists of the described MRI processing pipeline and the functional connectome-based predictive model. It is available as source code at https://github.com/spisakt/RPN-signature. As the software follows the Brain Imaging Data Structure (BIDS)47 and the BIDS-App specification, it provides a standard command line interface and relies on Docker-technology. The docker image is deposited on Docker Hub: (https://cloud.docker.com/repository/docker/tspisak/rpn-signature) and does not depend on any software outside the container image. This, together with the fully transparent continuous integration-based development and automated tagging and versioning, enhances software availability and supports reproducibility of RPN-signature results.

846 enhances software availability and supports reproducibility of RPN-signature results.

Field-specific reporting

Please select the one below that is the best fit for your research. If you are not sure, read the appropriate sections before making your selection.

☒ Life sciences ☐ Behavioural & social sciences ☐ Ecological, evolutionary & environmental sciences

For a reference copy of the document with all sections, see nature.com/documents/nr-reporting-summary-flat.pdf

Life sciences study design

All studies must disclose on these points even when the disclosure is negative.

Sample size	Sample size for Study 1 was determined as described in Zunhammer, M. et al. Combined glutamate and glutamine levels in pain-processing brain regions are associated with individual pain sensitivity. Pain 157, 2248–2256 (2016).<br>Sample sizes for Study2 and Study 3 were determined a-priory based on the expected prediction accuracy provided by nested cross-validation in Study 1 and were pre-registered before acquisition (http://osf.io/buqt7).
Data exclusions	25 participants were excluded from the total of 116 recruited participants due to extreme QST values or high in-scanner motion.<br>See Supplementary Table S2 for details.
Replication	All the presented results all fully and easily replicable given the data and software deposited at public repositories, as listed above.<br>A remarkable reproducibility on new datasets is expected as the predictive model trained in Study 1 turned out to generalize well to the independent validation Studies 2 and 3 (conducted in different centers and with different equipment by different research staff).
Randomization	No experimental groups were used, as the predictive model targets a continuous covariate (individual sensitivity to pain).<br>Therefore no randomization was used. During model training a leave-one-participant-out nested cross validation was performed to improve generalizability and attenuate overfitting.
Blinding	The experimental design required no blinding. MRI measurements, serving as the predictive feature set, were always preceding the acquisition of the target data (QST pain sensitivity measurements), therefore neither the researchers, nor the participants were informed about the actual individual pain sensitivities during image acquisition.

Reporting for specific materials, systems and methods

We require information from authors about some types of materials, experimental systems and methods used in many studies. Here, indicate whether each material, system or method listed is relevant to your study. If you are not sure if a list item applies to your research, read the appropriate section before selecting a response.

Materials & experimental systems

n/a	Involved in the study
☒	☐ Antibodies
☒	☐ Eukaryotic cell lines
☒	☐ Palaeontology
☒	☐ Animals and other organisms
☐	☒ Human research participants
☒	☐ Clinical data

Methods

n/a	Involved in the study
☒	☐ ChIP-seq
☒	☐ Flow cytometry
☐	☒ MRI-based neuroimaging

Human research participants

Policy information about studies involving human research participants

Population characteristics	Study 1: N=39, Age(mean± sd)=26.1± 3.9, Sex(%female): 37%<br>Study 2: N=48, Age(mean± sd)=24.9± 3.5, Sex(%female): 54%<br>Study 3: N=29, Age(mean± sd)=24.8± 3.1, Sex(%female): 53%
Recruitment	Recruitment was done by advertisements at Universities. Recruitment and reimbursement policies varied across centers; participants received 20 €/h in Studies 1 and 2 and no reimbursement in Study 3.<br>While volunteer bias might be present in the data, the potential volunteers were informed that the QST-based pain-threshold measurement is only slightly painful (being stopped at the onset of pain). Therefore volunteer-bias is expected to be much lower than in typical pain studies. Moreover, the developed predictive model allows for non-invasive, pain-free assessment of pain thresholds, thus further mitigating issues of volunteer-bias typical in pain research.
Ethics oversight	The study was conducted in accordance with the Declaration of Helsinki and approved by the local or national ethics committees (Register Numbers: 4974-14, 18-8020-BO and 057617/2015/OTIG at the Ruhr University Bochum, University Hospital Essen and ETT TUKEB Hungary, respectively.) All participants gave written informed consent before testing.

Note that full information on the approval of the study protocol must also be provided in the manuscript.

Magnetic resonance imaging

Experimental design

Design type	resting-state design (pain-free)
Design specifications	Scan length was 8min 37sec, 12min 11 sec and 10 min in Studies 1, 2 and 3, respectively.
Behavioral performance measures	No behavioral data was recorded during scanning.

Acquisition

Imaging type(s)	functional
Field strength	3T
Sequence & imaging parameters	Study 1: GE EPI, FOV: 240x240x132mm, matrix: 80x80, slice thickness: 3mm, interleaved slices, TE=35ms, TR=2500ms, flip angle=90°<br>Study 2: GE EPI, FOV: 230x230x132mm, matrix: 94x94, slice thickness: 3mm, interleaved slices, TE=35ms, TR=2520ms, flip angle=90°<br>Study 3: GE EPI, FOV: 288x288x132mm, matrix: 96x96, slice thickness: 3mm, interleaved slices, TE=27ms, TR=2500ms, flip angle=90°
Area of acquisition	whole brain scan

Diffusion MRI ☐ Used ☒ Not used

Preprocessing

Preprocessing software	Prerocessing MRI data, training and validating model: "PUMI" in-house software library system (https://github.com/spisakt/PUMI, v0.1.1a), "RPN-signature" in-house predictive modeling pipeline (https://github.com/spisakt/RPN-signature, v0.1.5).<br>The processing pipeline utilizes FSL (v6.0.1), AFNI (AFNI_17.3.03), nipype (v1.1.9), ANTs (v2.2.0.dev62-g074d4), python packages: numpy=1.15.4, scipy=1.1.0, scikit-learn=0.19.1, matplotlib=2.2.2, pandas=0.23.4, libxml2=2.9.8, libxslt=1.1.32, graphviz=2.40.1, traits=4.6.0, statsmodels=0.9.0, bids==0.0 nilearn==0.5.0 seaborn==0.9.0.<br><br>The full analysis pipeline is released as a BIDS-app and conserved into the Docker image tspisak/rpn-signature:v0.1.5 deposited on dockerhub: https://hub.docker.com/r/tspisak/rpn-signature.<br><br>For pre-processing parameters, see the source code of PUMI v0.1.1.a (https://github.com/spisakt/PUMI) and RPN-signature v0.1.5 (https://github.com/spisakt/RPN-signature).<br><br>Installation and usage information is available on the project webpage: https://spisakt.github.io/RPN-signature
Normalization	Anatomical data was normalized with ANTs, see the source code of PUMI v0.1.1.a (https://github.com/spisakt/PUMI) or https://gist.github.com/spisakt/0caa7ec4bc18d3ed736d3a4e49da7415 for parameters.<br>Functional data was co-registered to the anatomical image with FSL Flirt Boundary-based registration (BBR). Please refer to the source code of PUMI v0.1.1.a (https://github.com/spisakt/PUMI) for more details.<br>Analysis was done in native functional space by brain-atlas individualization.

Normalization template	The 1mm-resolution MNI152 Template was used.
Noise and artifact removal	Realignment-based motion correction was performed with FSL mcflirt. The resulting six head motion estimates (3 rotations, 3 translations), their squared versions, their derivates and the squared derivates (known as the Friston-24-expansion) was calculated and saved for nuisance correction. Additionally, head motion was summarised as frame-wise displacement (FD) timeseries, according to Power's method, to be used in data censoring and exclusion. After motion-correction, outliers (e.g. motion spikes) in time series data were attenuated using AFNI despike. The union of the eroded white-matter maps and ventricle masks were transformed to the native functional space and used for extracting noise-signal for anatomical CompCor correction. In a nuisance regression step, 6 CompCor parameters (the 6 first principal components of the noise-region timeseries), the Friston-24 motion parameters and the linear trend were removed from the timeseries data with a general linear model. On the residual data, temporal bandpass filtering was performed with AFNI's 3DBandpass to retain the 0.008-0.08Hz frequency band.
Volume censoring	The prior use of AFNI's despike is expected to attenuate aliasing of residual motion artefacts into the neighbouring time-frames during bandpass filtering. To further attenuate the impact of motion artefacts, potentially motion-contaminated time-frames, defined by a conservative FD>0.15mm threshold, were dropped from the data (known as "scrubbing" the data). Participants were excluded from further analysis if the mean FD exceeded 0.15mm, or when more then 30% of frames were "scrubbed".

Statistical modeling & inference

Model type and settings	Predictive model implemented in scikit-learn, consisting of robust feature scaling (removes the median and scales with data quantiles), pre-selection of features, selecting the K "best" features with strongest relationships to the target variable and an Elastic Net regression model (a linear model with combined L1 and L2-norms as regulariser). Free hyperparameters of the machine learning pipeline were the number of pre-selected features (K), the ratio of the L1/L2-regularization and the weight (alpha) of regularisation. Hyperparameters were optimised with a grid-search procedure and negative mean squared error as cost function. Values for K ranged from 10 to 200 with increments of 5, and included [.1, .5, .7, .9, .95, .99, 793 .999] for the L1/L2 ratio [.001, .005, .01, .05, .1, .5] for alpha. Hyperparameter optimisation was performed in a leave-one-participant-out cross validation (internal validation phase). Cross- validation incorporated the complete machine-learning pipeline to avoid introducing dependencies between the training and test samples. Note that fMRI preprocessing was independent between subjects, thus not included in the cross-validation.
Effect(s) tested	Prediction accuracy in Samples 2 and 3 was tested with permutation tests.

Specify type of analysis: ☐ Whole brain ☒ ROI-based ☐ Both

Anatomical location(s): MIST multi-resolution functional parcellation.

Statistic type for inference (See Eklund et al. 2016)	Does not apply.
Correction	No mass-univariate analysis.

Models & analysis

n/a	Involved in the study
☐	☒ Functional and/or effective connectivity
☐	☒ Graph analysis
☐	☒ Multivariate modeling or predictive analysis

Functional and/or effective connectivity	partial correlation
Graph analysis	Nodal summaries of predictive connectivity weight (sum of the weight of all links of a node) were used to aid interpretation.
Multivariate modeling and predictive analysis	Whole-brain atlas-based resting-state functional connectivity data of study 1 (N1=35, after all exclusions, Supplementary Table S2) was used as the input feature-space (P=7503 features per participant) to predict individual pain sensitivity scores, leading to a "large P — small N" setting. Feature selection was performed by pre-selection of features (selecting the K "best" features with strongest relationships to the target variable) and an Elastic Net regression model (a linear model with combined L1 and L2-norms as regulariser). Target variable was the QST-based composite pain sensitivity score, as previously defined in (Zunhammer, M. et al. Combined glutamate and glutamine levels in pain-processing brain regions are associated with individual pain sensitivity. Pain 157, 2248–2256 2016).
